# A new species of *Pristimantis* (Anura: Strabomantidae) from white-sand forests of central Amazonia, Brazil

**DOI:** 10.7717/peerj.15399

**Published:** 2023-06-06

**Authors:** Alexander Tamanini Mônico, Miquéias Ferrão, Jiří Moravec, Antoine Fouquet, Albertina P. Lima

**Affiliations:** 1 Programa de Pós-Graduação em Biologia (Ecologia), Instituto Nacional de Pesquisas da Amazônia, Manaus, Amazonas, Brazil; 2 Museum of Comparative Zoology, Harvard University, Cambridge, MA, United States of America; 3 Department of Zoology, National Museum, Cirkusová, Prague, Czech Republic; 4 Laboratoire Evolution et Diversité Biologique, Université Paul Sabatier, Toulouse, France

**Keywords:** Amphibia, Advertisement call, Campinarana, Integrative taxonomy, Phylogeny, *Pristimantis unistrigatus* species group

## Abstract

The white-sand ecosystems in the Solimões-Negro Interfluve are among the less studied in Amazonia. Recent herpetological surveys conducted west of Manaus, Brazil (central Amazonia) indicate that white-sand forests host a unique anuran fauna comprising habitat specialized and endemic species. In the present study we describe a new species of rain frog belonging to the *Pristimantis unistrigatus* species group from the white-sand forest locally called “*campinarana*” (thin-trunked forests with canopy height below 20 m). The new species is phylogenetically close to rain frogs from western Amazonian lowlands (*P. delius*, *P. librarius, P. matidiktyo* and *P. ockendeni*). It differs from its closest relatives mainly by its size (male SVL of 17.3–20.1 mm, *n* = 16; female SVL of 23.2–26.5 mm, *n* = 6), presence of tympanum, tarsal tubercles and dentigerous processes of vomers, its translucent groin without bright colored blotches or marks, and by its advertisement call (composed of 5–10 notes, call duration of 550–1,061 ms, dominant frequency of 3,295–3,919 Hz). Like other anuran species recently discovered in the white-sand forests west of Manaus, the new species seems to be restricted to this peculiar ecosystem.

## Introduction

The genus *Pristimantis* Jiménez de la Espada, 1870 is the most species-rich among vertebrates. Nevertheless, its total species diversity remains highly underestimated as suggested by the numerous new species described each year (*e.g.*, De Oliveira et al., 2020; Ron et al., 2020; Gete, Buckely & Ron, 2021; Fouquet et al., 2022a; Ortega, Brito & Ron, 2022; Roberto et al., 2022). Since 2020 around 60 species of *Pristimantis* were described (Frost, 2023). The Andes is the most species-rich region for *Pristimantis* (Pinto-Sanchez et al., 2012), hosting more than 60% of the nominal species of the genus (Meza-Joya & Torres, 2015). Many other species are distributed throughout Amazonia, and the rest occurs in the Cerrado, Atlantic Forest, Pantepui and in Trans-Andean forest areas (Frost, 2023). This species-richness disparity is at least partially explained by the evolutionary history of the genus that started to diversify in the Andes and dispersed many times to Amazonian lowlands (Mendoza et al., 2015) with subsequent dispersals back to the Andes (Fouquet et al., 2022b). However, such disparity is also a consequence of the large amount of poorly sampled areas in Amazonia, where many undescribed species of *Pristimantis* occur (*e.g*., Vacher et al., 2020; Fouquet et al., 2022a). Finally, the low number of taxonomists working in lowland Amazonia also contributes to this knowledge gap (Melo-Sampaio, Ferrão & Moraes, 2021).

Amazonia is composed mostly of tropical ombrophilous rainforests (Veloso, Rangel Filho & Lima, 1991), but many other types of habitats exist due to variation in edaphic, hydrological and climatic conditions (Prance, 1979; Terborgh & Andresen, 1998), notably the white-sand ecosystem (WSE). It is characterized by grasslands, shrublands or forests with low-stature canopies on nutrient-poor sandy soils (Eiten, 1978). In central Amazonia, the WSE can be subdivided in two main types (Adeney et al., 2016): *campina* (open grasslands and shrublands with canopy height below 10 m) and *campinarana* (closed-canopy, thin-trunked forests with canopy height below 20 m). Although still poorly documented (Adeney et al., 2016), recent studies have indicated that WSE harbors a high proportion of specialized species for several biological groups, like birds (*e.g.,* Almeida, 2010; Borges et al., 2016; Matos et al., 2016; Capurucho et al., 2020), snakes (*e.g*., Fraga et al., 2018) and plants (*e.g.*, Fine & Baraloto, 2016; Vicentini, 2016; Guevara et al., 2016; Costa et al., 2020). For anurans, data collection is still incipient, albeit WSE specialization has been hypothesized for three hylid frogs (Carvalho et al., 2018; Ferrão et al., 2019; Ferrão et al., 2022).

Anuran sampling in Amazonia is concentrated near urban areas, navigable rivers, roads and highways (Jenkins et al., 2015; see map in Vacher et al., 2020). This is especially true for the Negro-Solimões Interfluve (NSI), a region with the largest amount of WSE (see Adeney et al., 2016) and only a few documented anuran assemblages (Neckel-Oliveira & Gordo, 2004; Menin et al., 2017; Moraes et al., 2022a; Moraes et al., 2022b). Even regions geographically close to the largest city in Brazilian Amazonia (Manaus) and regions easy to access remain poorly sampled and studied. However, this picture is changing. The Reserva do Desenvolvimento Sustentável do Rio Negro (RDS Rio Negro)—a NSI reserve covered mainly by a mosaic of dense forests and WSE patches lying ca. 100 km west of Manaus—has recently become a research center for biodiversity studies focussing on this ecosystem. The first attempt to document the anuran communities of the RDS Rio Negro has notably resulted in the rediscovery of an overlooked spiny-backed treefrog (Ferrão et al., 2019) and the description of a new snouted treefrog (Ferrão et al., 2022).

Herpetological surveys conducted in the RDS Rio Negro and nearby WSE patches in 2018 and 2020 resulted in the discovery of an unknown *Pristimantis* species associated with *campinarana*. The external morphology and the advertisement call of this species indicated that it represents an unnamed species and preliminary molecular comparisons confirmed this assumption. Herein, we use an integrative taxonomy approach and describe this new species of *Pristimantis* as well as its phylogenetic position, geographic distribution and natural history.

## Materials & Methods

### Sampling

Twenty-two adults and two juveniles of the new species were manually collected in two localities in the municipality of Iranduba, state of Amazonas, Brazil. Among them, 20 specimens were collected at the Ramal Nova Esperança, Km 20 of the AM-070 Highway (3°09′14.5 ″S, 60°13′59.4″W, 83 m elevation) on 13 December 2020, and four specimens at the RDS Rio Negro (3°03′42.0″S, 60°45′02.1″W, 61 m elevation) on 14 September 2018. Specimens were anaesthetized and killed with topic 5% lidocaine. Muscle or liver tissue was preserved in 100% ethanol for posterior genetic analysis, whereas the specimens were fixed in 10% formalin and subsequently preserved in 70% ethanol. Specimens were sexed by the presence of vocal slits exclusively present in males and internally by the condition of the gonads. All males used in the type series were found calling, which ensures their reproductive status as adults, while gravid females (*n* = 4) and specimens with large SVL were also considered adults. Vouchers were deposited in the herpetological collection of the Instituto Nacional de Pesquisas da Amazônia—INPA-H (Manaus, Brazil) and Museu Paraense Emílio Goeldi—MPEG (Belém, Brazil). Protocols of collection and animal care follow the Brazilian Federal Council for Biology resolution number 148/2012 (Conselho Federal de Biologia—CFBio, 2012) and the Ethics Committee on the Use of Animals of the Instituto Nacional de Pesquisas da Amazônia—CEUA-INPA (Process n° 35/2020, SEI 01280.001134/2020-63). Specimens were collected under collection permit number 73647-3 issued by the Centro Nacional de Pesquisa e Conservação de Répteis e Anfíbios of the Instituto Chico Mendes de Conservação da Biodiversidade—ICMBio.

Advertisement calls of nine males of the new species were recorded at the Ramal Nova Esperança on 13 December 2020. Recordings were made with a Sennheiser K6/ME66 unidirectional microphone (Sennheiser, Germany) coupled to a digital recorder Marantz PMD660 (Marantz, Japan). Air temperatures (24.6–26.2 °C) and humidity (86–93%) during call recording were measured with a thermohygrometer Incoterm 7663.02.0.00. Each calling male was recorded for three minutes using frequency rate of 44 kHz and 16 bits of resolution in the mono pattern. Recordings were deposited in the Fonoteca Neotropical Jacques Vielliard (FNJV) of the Universidade de Campinas (UNICAMP), Campinas, Brazil under access number FNJV 59105–59115.

### Sequencing and phylogenetic analyses

Genomic DNA was extracted from tissues of five specimens from Ramal Nova Esperança and three from RDS Rio Negro using the kit PureLink™ Genomic DNA (Invitrogen by Thermo Fisher Scientific, Carlsbad, CA, USA). Fragments of two mitochondrial (16S rRNA and Cytochrome C Oxidase sub-unit 1—COI) and a nuclear gene (Recombination Activating 1—RAG1) were amplified through polymerase chain reaction (PCR) following the protocol described in Mônico et al. (2022). The 16S was amplified using primers 16Sar (5′-CGCCTGTTTATCAAAAACAT-3′) and 16Sbr (5′-CCGGTCTGAACTCAGATCACGT-3′) (Palumbi, 1996), COI using primers Chmf4f (5′-TYTCWACWAAYCAYAAAGAYATCGG-3′) and Chmr4r (5′-ACYTCRGGRTGRCCRAARAATCA-3′) (Che et al., 2012) and RAG1 using primers R182 (5′-GCCATAACTGCTGGAGCATYAT-3′) and R270 (5′-AGYAGATGTTGCCTGGGTCTTC-3′) (Heinicke, Duellman & Hedges, 2007). Amplicons were sequenced in an ABI PRISMI 3130XL (Thermo Fisher) using the forward and reverse primers of each gene. Sequences were manually edited with Geneious Pro 5.4.6 (Biomatters Ltd.), then subjected to BLAST (https://blast.ncbi.nlm.nih.gov/Blast.cgi; Altschul et al., 1997) to compare with sequences of other *Pristimantis*. Newly generated sequences were deposited in GenBank. Accession numbers are available in Appendix 1.

According to BLAST, sequences of the new species were highly similar to species currently assigned to the *Pristimantis unistrigatus* species group. To infer the phylogenetic relationships among the new species and its close relatives, newly generated sequences were inserted into a data set retrieved from GenBank containing selected homologous sequences (Appendix 1). Sequence selection in GenBank was focused on specimens of the *P. unistrigatus* species group from the Andes, Pantepui and Amazonian lowlands. Additionally, sequences of two species of the genus *Oreobates* were retrieved to root the tree. In total, 265 sequences (16S = 136; COI = 70; RAG1 = 59) corresponding to 137 specimens were selected. We aligned sequences of each gene using MAFFT online server (https://mafft.cbrc.jp/alignment/server/) with default parameters, except by the use of the E-INS-i strategy for the 16S and G-INS-i strategy for protein-coding genes (Katoh & Standley, 2013). The final matrix was concatenated in Mesquite (Maddison & Maddison, 2021) and composed of 137 terminals with 1,827 bp (16S = 561 pb; COI = 636 pb; RAG1 = 630 pb).

Best-fit evolutionary models and partition schemes were determined through ModelFinder (Kalyaanamoorthy et al., 2017) using seven initial partitions: one for the 16S and one for each codon of protein-coding genes. The best evolutionary models for partitions in the concatenated matrix were: TIM2 + F + R5 for 16S, GTR + F + I +G4 for COI 1st and RAG 3rd codons, HKY + F +G4 for COI 2nd, RAG 1st and 2nd codons, and GTR + F +ASC + G4 for COI 3rd position. Phylogenetic relationships were reconstructed using Maximum Likelihood inference (ML). The ML tree was inferred with IQTREE (Nguyen et al., 2015) as implemented in the webserver (http://iqtree.cibiv.univie.ac.at; Trifinopoulos et al., 2016). Clade support was estimated with 10,000 ultrafast bootstrap replications (Hoang et al., 2018), 1,000 maximum iterations, and a minimum correlation coefficient of 0.99. We calculated pairwise genetic distances (*p*-distance and Kimura-two-parameter distance; Kimura, 1980) among the populations of new species and close relatives using MEGA 11 (Tamura, Stecher & Kumar, 2021). Genetic distances were calculated using pairwise deletion.

### Morphology

Twenty five morphometric measurements were taken from 16 adult males and six adult females of the new species following Duellman & Lehr (2009) (eye diameter—ED, eye-nostril distance—EN, foot length—FL, interorbital distance—IOD, internarial distance—IND, head length—HL, head width—HW, snout-vent length—SVL, tibia length—TL, and tympanum diameter—TD), Caldwell, Lima & Keller (2002) (forearm length—FAL, hand length—HAND, snout length—SL, disc width of Finger III—WFD), Heyer et al. (1990) (tarsus length—TAL, thigh length—THL, upper arm length—UAL), Lima, Sanchez & Souza (2007) (hand length from proximal edge of palmar tubercle to tip of Finger I—HANDI, Finger II—HANDII, and Finger IV—HANDIV) and Mônico et al. (2022) (foot length from proximal edge of outer metatarsal tubercle to tip of Toe I—FLI, Toe II—FLII, Toe III—FLIII and Toe V—FLV, and disc width of Toe IV—WTD). Measurements were taken to the nearest 0.01 mm using a Leica stereomicroscope (model S8APO) coupled to a Leica DFC295 camera, except for SVL, measured to the nearest 0.01 mm with a digital caliper. Raw data are provided in Table S1.

Format of the description and terminology of morphological characters follow Kok & Kalamandeen (2008), Duellman & Lehr (2009), and Kok, Means & Bossuyt (2011). Color in life was described based on photographs taken in the field, following the color catalog provided by Köhler (2012).

### Bioacoustics

Bioacoustic variables were analyzed with Raven Pro 1.6 (Bioacoustics Research Program, 2014) with the following configuration: window = Blackman, Discrete Fourier Transform = 2,048 samples and 3dB filter bandwidth = 80.0 Hz. The following temporal and spectral traits were measured: call duration—CD, number of notes per call—NN, note duration—ND, inter-note interval—SBN, and minimum—LF, maximum—HF and dominant frequency—DF. Inter-call interval was not measured because it is usually affected by microclimatic conditions at the time of recording (*i.e*., on rainy days, males call more often in a short period of time than on days without rain) and poorly informative. Dominant frequency was measured using the *Peak frequency* function; maximum and minimum frequencies were measured 20dB below the peak frequency to avoid background noise interference. Call description follows the call centered approach of Köhler et al. (2017). Spectrogram and oscillogram were generated in R environment (R Core Team, 2019) through the ‘*seewave*’ package 2.0.5 (Sueur, Aubin & Simonis, 2008) using a Hanning window, 256 points of resolution (Fast Fourier Transform) and an overlap of 85%. Bioacoustic raw data are provided in Tables S2.

### Nomenclatural acts

The electronic version of this article in Portable Document Format (PDF) will represent a published work according to the International Commission on Zoological Nomenclature (ICZN), hence the new names contained in the electronic version are effectively published under that Code from the electronic edition alone. This published work and the nomenclatural acts it contains have been registered in ZooBank, the online registration system for the ICZN. ZooBank LSIDs (Life Science Identifiers) can be resolved and the associated information viewed through any standard web browser by appending the LSID to the prefix http://zoobank.org/. The LSID for this publication is: urn:lsid:zoobank.org:pub:F8ED54F6-9E18-49BD-8178-659BDFB79C65. The online version of this work is archived and available from the following digital repositories: PeerJ, PubMed Central and CLOCKSS.

## Results

### Phylogenetic relationships and genetic distances

**Figure 1:**
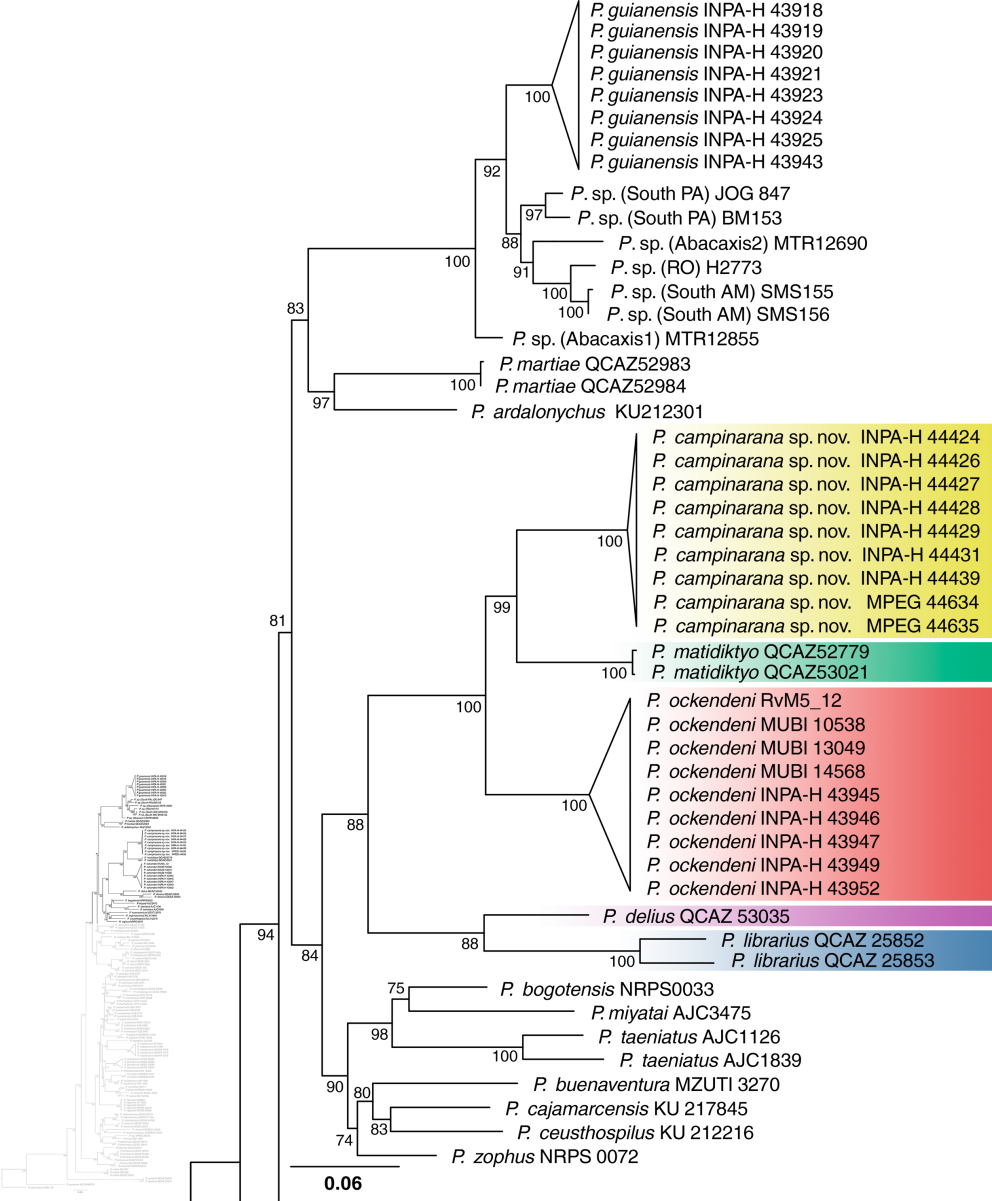
Part of the phylogenetic tree showing the position of *Pristimantis campinarana* sp. nov. Maximum likelihood tree inferred with 16S, COI and RAG1. Non-parametric ultrafast bootstrap support is shown near the nodes. The species name is preceded by the specimen voucher number. The full phylogenetic tree is presented in Appendix 2.

**Figure 2:**
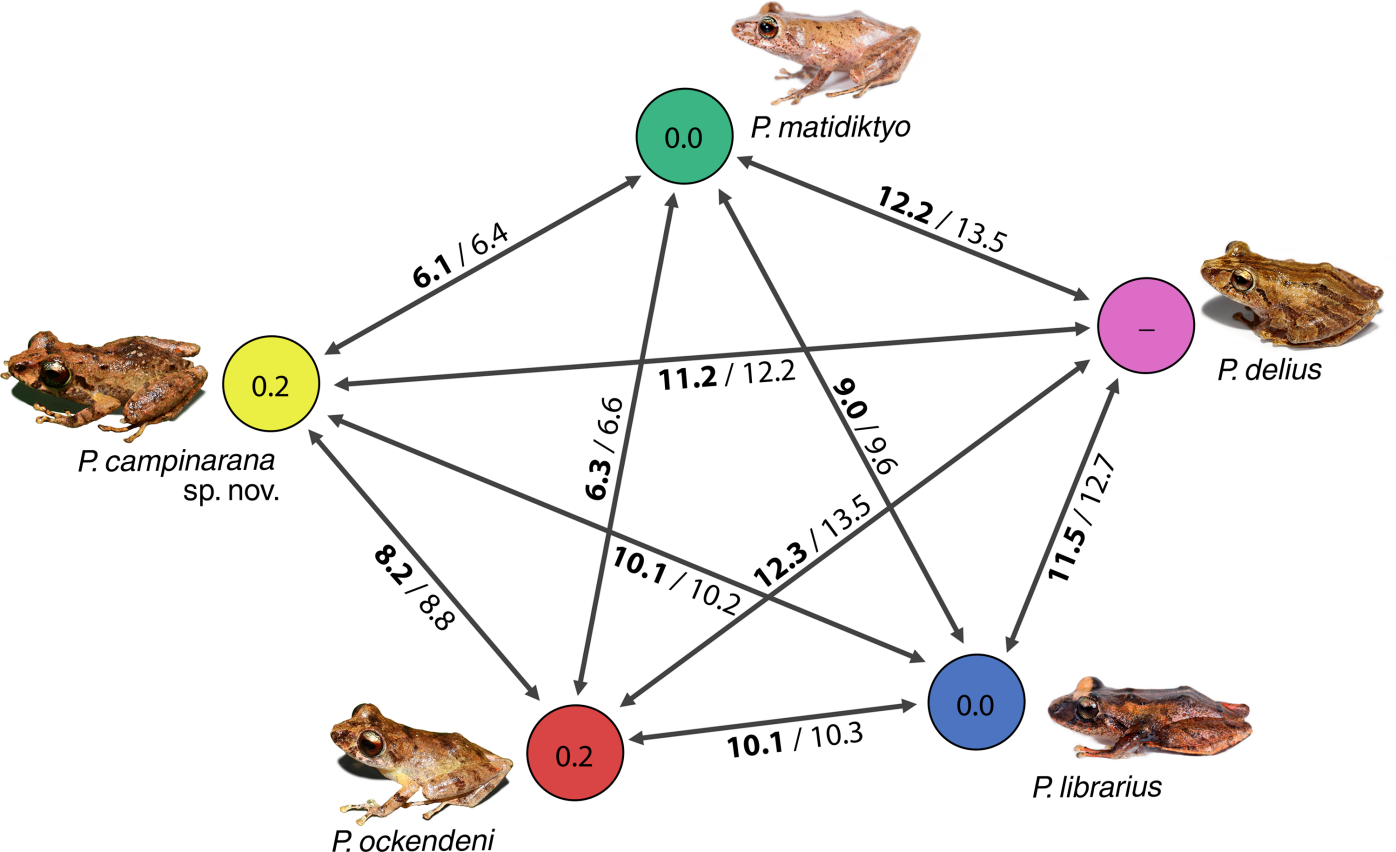
Genetic distances between *Pristimantis campinarana* sp. nov. and most closely related species (in percentage and based on 554 bp of 16S). Mean interspecific distances are shown above or below arrows; *p*-distances are presented in bold numbers and followed by Kimura-2-parameters distance. Intraspecific p-distance is shown inside the circles. Photographs: S.R. Ron, PUC Ecuador, 2021 (*P. delius*, *P. librarius* and *P. matidiktyo*) and A.T. Mônico (*P. campinarana* sp. nov. and *P. ockendeni*).

Individuals of the new species show low intraspecific genetic distances between populations (16S p-distance = mean 0.2%, maximum 0.4%). The new species is nested within a strongly supported clade grouping *Pristimantis matidiktyo* Ortega-Andrade & Valencia, 2012 and *P. ockendeni* (Boulenger, 1912) (Fig. 1, Appendix 2). This clade is sister to the clade formed by *P. delius* (Duellman & Mendelson III, 1995) and *P. librarius* (Flores & Vigle, 1994). Among the species mentioned above, mean interspecific p-distances range from 6.1 to 11.2% (Fig. 2). All these species occur in western Amazonian lowlands.

### Taxonomic account

### *Pristimantis campinarana* sp. nov.

LSID: urn:lsid:zoobank.org:act:61E81E96-5309-4FA0-AE8A-B34636CD5616

Figures 3–6, 9.

*Pristimantis ockendeni*: Lima et al. (2021).

### Holotype

INPA-H 44426 (field number APL 23164), adult male, collected at Ramal Nova Esperança, Km 20 of the AM-070 Highway, municipality of Iranduba, state of Amazonas, Brazil (3°09′14.5″S, 60°13′59.4″W, 83 m elevation), on 13 December 2020 by A. T. Mônico and E. D. Koch.

### Paratypes

Eighteen adult specimens collected at the same locality as the holotype on 12–19 December 2020 by A. T. Mônico, I. Y. Fernandes and E. D. Koch: twelve males INPA-H 44424, INPA-H 44427–29, INPA-H 44431–35 (field numbers APL 23162, 23165–67, 23169–72 and 23175 respectively) and MPEG 44637, MPEG 44639 and MPEG 44641 (field numbers APL 23181, 23183 and 23185, respectively); and six females INPA-H 44425, INPA-H 44436 and INPA-H 44437 (field numbers APL 23163, 23176 and 23177, respectively) and MPEG 44636, MPEG 44638 and MPEG 44640 (field numbers APL 23179, 23182 and 23184, respectively), and three adult males collected at Reserva do Desenvolvimento Sustentável do Rio Negro, municipality of Iranduba, state of Amazonas, Brazil(3°03′42.0″S, 60°45′02.1″W, 61 m elevation): MPEG 44634–35 and INPA-H 44439 (field numbers APL 22250–52, respectively), on 14 September 2018 by M. Ferrão, A. S. Ferreira and J. Moravec.

### Referred material

Four specimens collected in the municipality of Iranduba, state of Amazonas, Brazil. One juvenile (INPA-H 44430, field number APL 23168), same data as the holotype. One juvenile (INPA-H 44440, field number APL 22253) and one adult male (INPA-H 44441, field number APL 22254), same data as the paratype INPA-H 44439. One adult male (INPA-H 44438, field number ATM 013) collected at the surroundings of Reserva do Desenvolvimento Sustentável do Rio Negro (3°06′36.5″S, 60°44′23.5″W), on 15 October 2022 by A. T. Mônico and I. Y. Fernandes. Two females (INPA-H 44698 and INPA-H 44699, field number ATM 49 and ATM 50, respectively) collected at Reserva do Desenvolvimento Sustentável do Rio Negro (2°55′16.6″S, 60°49′18.3″W), on 11 and 12 January 2023 by A. T. Mônico, I. Y. Fernandes, E. D. Koch, B. C. Martins, S. Dantas and A. P. Lima.

### Diagnosis

This new species is characterized by the following combination of characters: (1) dorsal skin shagreen; (2) tympanum visible, tympanic membrane poorly differentiated, tympanum diameter 29–40% of eye diameter and annulus partially visible externally; dark supratympanic band; (3) snout moderately long (SL 37–43% of HL), subacuminate in dorsal view and truncated in lateral view, loreal region concave, lips not flared; (4) upper eyelid tubercles present; with or without dark bar between the eyes, and one or two oblique black streaks below the eye; postrictal tubercles absent; cranial crests absent; three scapular tubercles, less visible in specimens having dark dorsal coloration; (5) nostril ovoid, slightly protuberant, directed laterally; internarial distance 73–87% of interorbital distance; (6) tongue ovoid, longer than wide; (7) dentigerous processes of vomers present, small, oblique and positioned posterior to level of choanae, one or two on each side, ill-defined; (8) males with vocal slits; vocal sac small, subgular; nuptial pads absent; (9) Finger II longer than I; finger discs small, rounded (Finger I and II) to expanded (Finger III and IV); (10) fingers without lateral fringes; (11) ulnar tubercles aligned, barely visible in fixed specimens; (12) tibia length 46–54% of SVL; (13) heel tubercle absent; tarsal tubercles aligned, small and barely visible; tarsal fold absent; (14) thenar tubercle ovoid; palmar tubercle bifid, twice the width of the thenar tubercle; (15) inner metatarsal tubercle ovoid; outer metatarsal tubercle small, longer than wide, less than 1/3 of the thenar tubercle; (16) toes III–V with lateral fringes, webbing basal between toes IV–V; Toe I smaller than Toe II, not reaching the edge of disc on Toe II; Toe V longer than Toe III; (17) belly skin weakly areolate, and ventral region of the femur areolate; (18) in life, groin translucent, without brightly colored blotches or marks; posterior surfaces of thighs uniformly brown; (19) in life, iris dichromatic, pale bronze upper and lower parts with dark brown vermiculation and broad median mahogany-red stripe through pupil; (20) SVL in adult males of 17.3–20.1 mm (*n* = 16) and in females of 23.2–26.5 mm (*n* = 6); and (21) advertisement call with 5–10 notes and average call duration of 694 ± 115 ms, inter-note interval of 82.7 ± 11 ms, minimum frequency of 2,260–3,176 Hz, maximum frequency of 3,756–5,280 Hz and dominant frequency of 3,295–3,919 Hz.

### Comparisons with other species

The new species is compared to all other currently recognized rain frogs of the *Pristimantis unistrigatus* species group occurring in Amazonian lowlands: *P. aaptus* (Lynch & Lescure, 1980); *P. academicus* (Lehr, Moravec & Gagliardi-Urrutia, 2010); *P. altamazonicus* (Barbour & Dunn, 1921); *P. brevicrus* (Andersson, 1945); *P. carvalhoi* (Lutz & Kloss, 1952); *P. crepitaculus* (Fouquet et al., 2022a; Fouquet et al., 2022b); *P. croceoinguinis* (Lynch, 1968); *P. delius* (Duellman & Mendelson III, 1995); *P. diadematus* (Jiménez de la Espada, 1875); *P. divnae* (Lehr & Von May, 2009); *P. espedeus* (Fouquet et al., 2013); *P. eurydactylus* (Hedges & Schlüter, 1992); *P. grandoculis* (Van Lidth de Jeude, 1904); *P. guianensis* (Mônico et al., 2022); *P. inguinalis* (Parker, 1940); *P. kichwarum* (Elmer & Cannatella, 2008); *P. librarius* (Flores & Vigle, 1994); *P. luscombei* (Duellman & Mendelson III, 1995); *P. martiae* (Lynch, 1974); *P. matidiktyo* (Ortega-Andrade & Valencia, 2012); *P. miktos* (Ortega-Andrade & Venegas, 2014); *P. ockendeni* (Boulenger, 1912); *P. orcus* (Lehr, Catenazzi & Rodríguez, 2009); *P. variabilis* (Lynch, 1968); and *P. ventrimarmoratus* (Boulenger, 1912).

*Pristimantis campinarana* sp. nov. differs from *P. aaptus, P. diadematus, P. divnae, P. espedeus* and *P. orcus* by having smaller male SVL of 17.3–20.1 mm [SVL 22.9 mm in *P. aaptus* (Lynch & Lescure, 1980); 20.0–27.4 mm in *P. diadematus* (Duellman & Mendelson III, 1995); 22.8–23.4 mm in *P. divnae* (Lehr & Von May, 2009); 20.7–24.8 mm in *P. espedeus* (Fouquet et al., 2013), and 20.0–25.1 mm in *P. orcus* (Lehr, Catenazzi & Rodríguez, 2009)] and from *P. academicus*, *P. carvalhoi*, *P. martiae* and *P. grandoculis* by larger male SVL [SVL 14.9 mm in *P. academicus* (Lehr, Moravec & Gagliardi-Urrutia, 2010); 13.5–14.8 mm *P. carvalhoi* (Lynch & Lescure, 1980; Duellman & Mendelson III, 1995); 14.7–17.9 mm in *P. grandoculis* (Fouquet et al., 2022a); 13.2–16.8 mm in *P. martiae* (Lynch, 1974)]; from *P. aaptus*, *P. altamazonicus*, *P. brevicrus*, *P. delius*, *P. diadematus, P. eurydactylus*, *P. miktos, P. ockendeni*, *P. orcus,* and *P. ventrimarmoratus* by smaller female SVL of 23.2–26.5 mm [SVL 29.9–34.8 mm in *P. aaptus* (Lynch & Lescure, 1980); 28.4–30.1 mm in *P. altamazonicus* (Ortega-Andrade et al., 2017); 27.2–35.0 mm in *P. brevicrus* (Ortega-Andrade et al., 2017); 30.9 mm in *P. delius* (Duellman & Mendelson III, 1995; Duellman & Lehr, 2009); 35.4–44.5 in *P. diadematus* (Duellman & Lehr, 2009); 29.4 mm in *P. espedeus* (Fouquet et al., 2013); 33.5–35.3 mm in *P. eurydactylus* (Hedges & Schlüter, 1992); 26.7–29.2 mm in *P. miktos* (Ortega-Andrade & Venegas, 2014); 30.4–30.6 mm in *P. ockendeni* (Mônico et al., 2022); 32.6–36.5 mm in *P. orcus* (Lehr, Catenazzi & Rodríguez, 2009), and 33.3–43.8 mm in *P. ventrimarmoratus* (Duellman & Lehr, 2009)].

*Pristimantis campinarana* sp. nov. can be easily distinguished from most of the lowland species (*n* = 16) by presence of vocal slits in males [absent in *P. altamazonicus* (Ortega-Andrade et al., 2017), *P. brevicrus* (Ortega-Andrade et al., 2017), *P. carvalhoi* (Duellman & Lehr, 2009), *P. croceoinguinis* (Lynch, 1968), *P. diadematus* (Duellman & Lehr, 2009), *P. divnae* (Lehr & Von May, 2009), *P. eurydactylus* (Hedges & Schlüter, 1992), *P. grandoculis* (Fouquet et al., 2022a), *P. miktos* (Ortega-Andrade & Venegas, 2014), *P. orcus* (Lehr, Catenazzi & Rodríguez, 2009), and *P. ventrimarmoratus* (Duellman & Lehr, 2009)], presence of eyelid tubercles [absent in *P. carvalhoi* (Duellman & Lehr, 2009), *P. delius* (Duellman & Mendelson III, 1995; Duellman & Lehr, 2009), *P. diadematus* (Duellman & Lehr, 2009), *P. lythrodes* (Lynch & Lescure, 1980), *P. variabilis* (Duellman & Lehr, 2009) and *P. ventrimarmoratus* (Duellman & Lehr, 2009)], presence of tympanum [absent in *P. brevicrus* (Ortega-Andrade et al., 2017), *P. carvalhoi* (Duellman & Lehr, 2009), *P. croceoinguinis* (Lynch, 1968; Duellman & Lehr, 2009), *P. grandoculis* (Fouquet et al., 2022a), *P. martiae* (Lynch, 1974) and *P. ventrimarmoratus* (Duellman & Lehr, 2009)], presence of dentigerous processes of vomers [absent in *P. delius* (Duellman & Mendelson III, 1995; Duellman & Lehr, 2009) and *P. guianensis* (Mônico et al., 2022)], and by presence of tarsal tubercles in males [absent in *P. diadematus* (Duellman & Lehr, 2009), *P. librarius* (Flores & Vigle, 1994), *P. lythrodes* (Lynch & Lescure, 1980), *P. kichwarum* (Elmer & Cannatella, 2008), *P. martiae* (Lynch, 1974; Duellman & Lehr, 2009), *P. matidiktyo* (Ortega-Andrade & Valencia, 2012) and *P. ventrimarmoratus* (Duellman & Lehr, 2009)].

Furthermore, the absence of brightly colored blotches or marks in the groin distinguishes the new species from *P. aaptus* (black groin; Lynch & Lescure, 1980), *P. academicus* (yellow groin; Lehr, Moravec & Gagliardi-Urrutia, 2010), *P. altamazonicus* (red to bright orange groin with black mottling; Ortega-Andrade et al., 2017), *P. brevicrus* (bluish white to yellowish white groin with black mottling; Ortega-Andrade et al., 2017), *P. carvalhoi* (yellow to yellowish white groin; Duellman & Lehr, 2009), *P. crepitaculus* (dark grey groin; Fouquet et al., 2022a), *P. croceoinguinis* (yellow or orange groin; Lynch, 1968; Elmer & Cannatella, 2008; Duellman & Lehr, 2009), *P. diadematus* (bluish white, yellowish tan or pink groin; Duellman & Lehr, 2009), *P. divnae* (yellow groin with brown marks; Lehr & Von May, 2009), *P. espedeus* (red-orange groin; Fouquet et al., 2013), *P. eurydactylus* (pale tan groin with dark brown vertical or diagonal bars; Hedges & Schlüter, 1992), *P. grandoculis* (dark grey groin; Fouquet et al., 2022a), *P. inguinalis* (bright yellow groin; Fouquet et al., 2013), *P. librarius* (reddish orange paler groin; Flores & Vigle, 1994; Elmer & Cannatella, 2008), *P. lythrodes* (black with yellowish white groin; Lynch & Lescure, 1980), *P. martiae* (pale brown groin; Lynch, 1974), *P. matidiktyo* (pale yellowish white groin; Ortega-Andrade & Valencia, 2012), *P. miktos* (yellowish-tan groin; Ortega-Andrade & Venegas, 2014), and *P. orcus* (black groin with white or whitish blue blotches; Lehr, Catenazzi & Rodríguez, 2009). Moreover, dichromatic iris differs *P. campinarana* sp. nov. from the species having monochromatic iris: *P. academicus* (golden to bronze iris; Lehr, Moravec & Gagliardi-Urrutia, 2010); *P. altamazonicus* (coppery red iris; Ortega-Andrade et al., 2017); *P. brevicrus* (coopery red to silver iris; Ortega-Andrade et al., 2017); *P. carvalhoi* (pale gray iris; Duellman & Lehr, 2009); *P. croceoinguinis* (gray to dull bronze iris; Lynch, 1968; Duellman & Lehr, 2009); *P. divnae* (silver iris; Lehr & Von May, 2009; *P. lythrodes* (grayish brown iris; Duellman & Lehr, 2009); *P. miktos* (deep orange iris; Ortega-Andrade & Venegas, 2014); *P. orcus* (dark gray to gold with cupper tint; Lehr, Catenazzi & Rodríguez, 2009); in *P. ventrimarmoratus* (pale bronze iris; Duellman & Lehr, 2009).

The advertisement call of *Pristimantis campinarana* sp. nov. is relatively similar with the calls of *P. espedeus*, P*. guianensis* and *P. ockendeni.* Nevertheless, calls of the new species differ in temporal and spectral characteristics [*P. campinarana* sp. nov.: call duration 694 ± 115 ms (550–1061 ms), inter-note interval 82.7 ± 11.9 ms (64–109 ms), and dominant frequency 3,587 ± 204 Hz (3,295–3,919 Hz) from *P. espedeus* (call duration 330 ms (240–500 ms), dominant frequency 2,700 Hz (2,680–2,840 Hz); Fouquet et al., 2013), *P. guianensis* (call duration 232 ± 42 ms (158–371 ms), inter-note interval 44 ± 5 ms (14–56 ms); Mônico et al., 2022) and *P. ockendeni* (dominant frequency 2,864 ± 202 Hz (2,519–3,143 Hz); Mônico et al., 2022)]. Furthermore, the advertisement call of *P. campinarana* sp. nov. easily differs from *P. inguinalis* and *P. orcus* by having multi-noted calls (single note in both mentioned species: Fouquet et al., 2013; López-Rojas et al., 2013).

### Description of holotype

**Figure 3:**
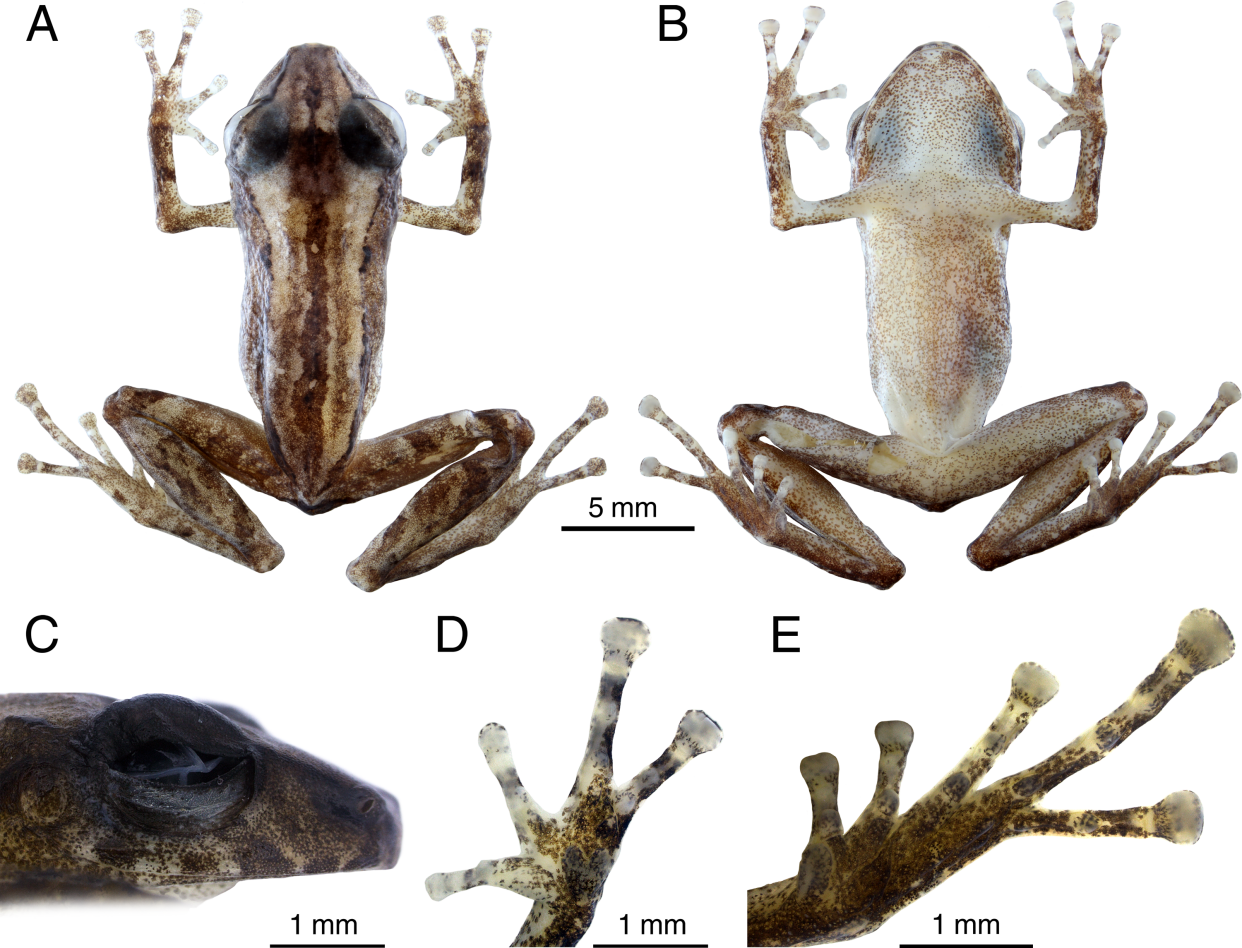
Preserved holotype (INPA-H 44426) of *Pristimantis campinarana* sp. nov. (A) Dorsal and (B) ventral views of body, (C) lateral view of head, (D) ventral view of hand, (E) ventral view of foot. Photographs: A.T. Mônico.

INPA-H 44426 (field number APL 23164). Morphometric measurements are presented in Table 1. An adult male (Fig. 3), SVL 19.1 mm; head slightly longer than wide (HL 103% of HW); head width 35.9% of SVL; head length 36.9% of SVL; cranial crest absent. Snout moderately long(SL 131% and 106% of EN and ED, respectively), subacuminate in dorsal view (Fig. 3A) and moderately truncated in lateral view (Fig. 3C); nostril ovoid, slightly protuberant, directed dorsolaterally; IND 87.3% of IOD; internarial region almost straight; *canthus rostralis* almost straight in dorsal view, slightly angular in profile; loreal region concave; lips not flared; one small tubercle on upper eyelid; interorbital region straight, IOD 32.1% of HW; eyes large (ED/TD = 2.7), pupil horizontally elliptical; supratympanic fold slightly distinct, extending from posterior margin of eyelid angling posteroventrally behind tympanic annulus; tympanum visible and rounded, TD 36.5% of ED; tympanic membrane poorly prominent, directed laterally; tympanic annulus poorly distinct, obscured dorsally by the supratympanic fold; one small postrictal tubercle, poorly visible; choanae of moderate sized, rounded to ovoid, not concealed by palatal sheath of maxilla; dentigerous processes of vomers present, with two ill-defined teeth, small, oblique and positioned posterior to level of choanae; tongue ovoid, longer than wide; short vocal slits, located in posterior half of mouth floor between tongue and margin of jaw; vocal sac small, simple and subgular.

Forearm shorter than hand (FAL 81.2% of HAND), notched posteriorly, nearly 78% free; three small ulnar tubercles ill-defined and aligned, almost indistinct after preservation; relative length of fingers I <II <IV <III (Fig. 3D); discs small and rounded on fingers I and II, expanded on fingers III and IV, with circumferential grooves; thenar tubercle poorly distinct, ovoid; palmar tubercle distinct, bifid; subarticular tubercles ill-defined, most prominent on fingers III and IV, rounded in dorsal and lateral view; small supernumerary tubercles present, but poorly visible; ventral pads well-defined on fingers III and IV.

Hindlimbs slender; tibia length 49% of SVL; heel without tubercles; tarsus with a row of small, poorly defined tubercles; tarsal fold absent; foot length 41% of SVL; relative length of toes I <II <III <V <IV (Toe III reaches the second subarticular tubercle of the Toe IV; Fig. 3E); toes with lateral fringes, more developed on toes III–V, webbing basal between toes IV–V; discs small and rounded on toes I and II, expanded on toes III–V; inner metatarsal tubercle large and ovoid, more than two times the size of ovoid outer metatarsal tubercle; subarticular tubercles large, protuberant, single, round on toes I–III and elliptical on toes IV and V; small supernumerary tubercles more visible on toes II-IV; ventral pads well-defined on toes III–V.

Dorsal skin shagreen (Fig. 3A), with longitudinal stripes and an irregular dorsolateral fold composed of spaced tubercles; small tubercles on scapular region; upper eyelid shagreen, with small tubercles; skin on flanks and chest smooth; skin on belly slightly areolate; upper and posterior surfaces of hindlimbs smooth, with small flat tubercles on thigh; dorsolateral folds absent; pectoral and discoidal folds not visible; cloaca protuberant, cloacal region without tubercles.

**Figure 4:**
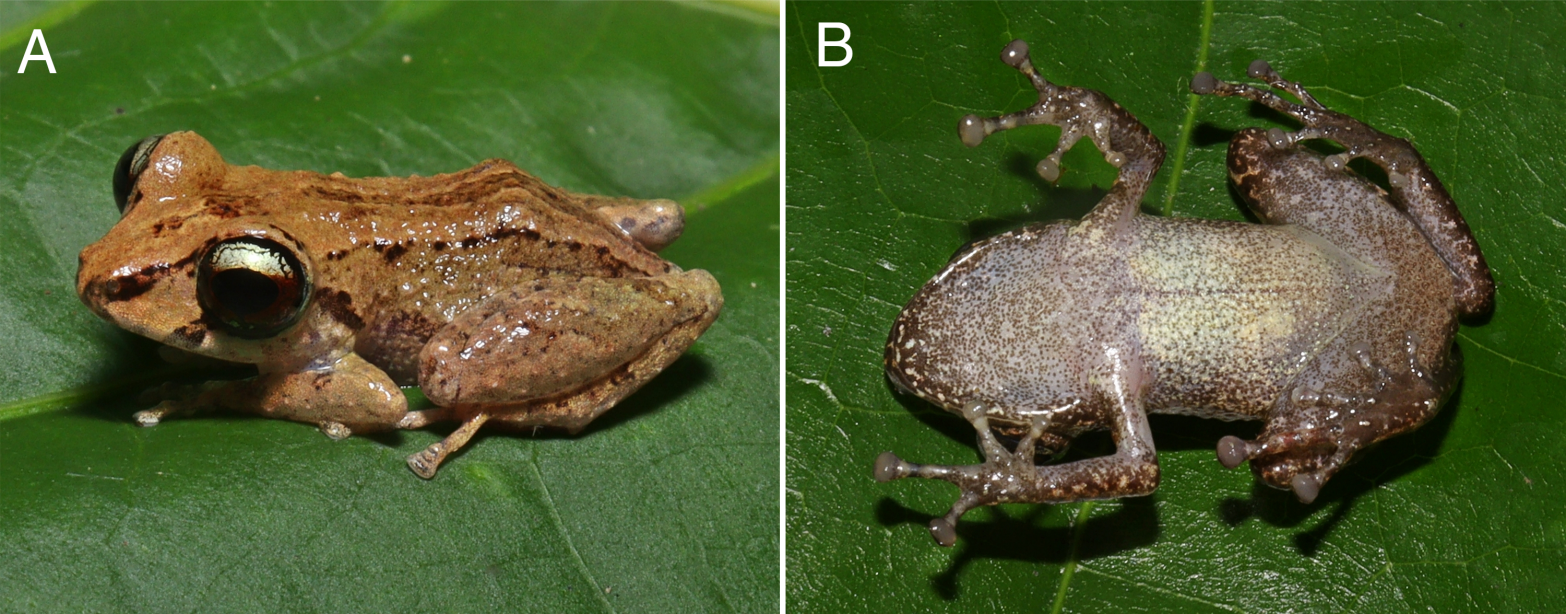
Coloration in life of the holotype (INPA-H 44426, SVL 19.1 mm) of *Pristimantis campinarana* sp. nov. (A) Dorsolateral and (B) ventral views of body. Photographs: A.T. Mônico.

In life, dorsum yellow ocher (color 14 by Köhler, 2012) with dark brown mid-dorsal stripe running from snout to cloaca. Canthal stripe present, black, running from the tip of snout to anterior margin of eyelid. Dorsolateral stripe present, irregular, formed by a series of small, dark brown dots and blotches, running from posterior eye to cloacal region (Fig. 4A). Upper lip with dark brown subocular and dark brown supratympanic bar. Dark brown transversal bars on forearms; light brown transversal bars on thigh and tibia. Posterior surfaces of thigs uniformly brown. Groin white, translucent, with sparse brown melanophores. Throat, chest, belly and ventral surfaces of legs white, translucent, densely covered by tiny brown melanophores (Fig. 4B). Iris pale bronze with dark brown vermiculation and broad horizontal median mahogany-red (color 34 by Köhler, 2012) stripe through pupil.

In alcohol, color pattern faded (Fig. 3), upper lip with five dark bars (Fig. 3C), bars on dorsal surfaces of forearms, thighs and tibias dark brown; venter whitish with dense dark brown melanophores.

### Variation in the type series

**Figure 5:**
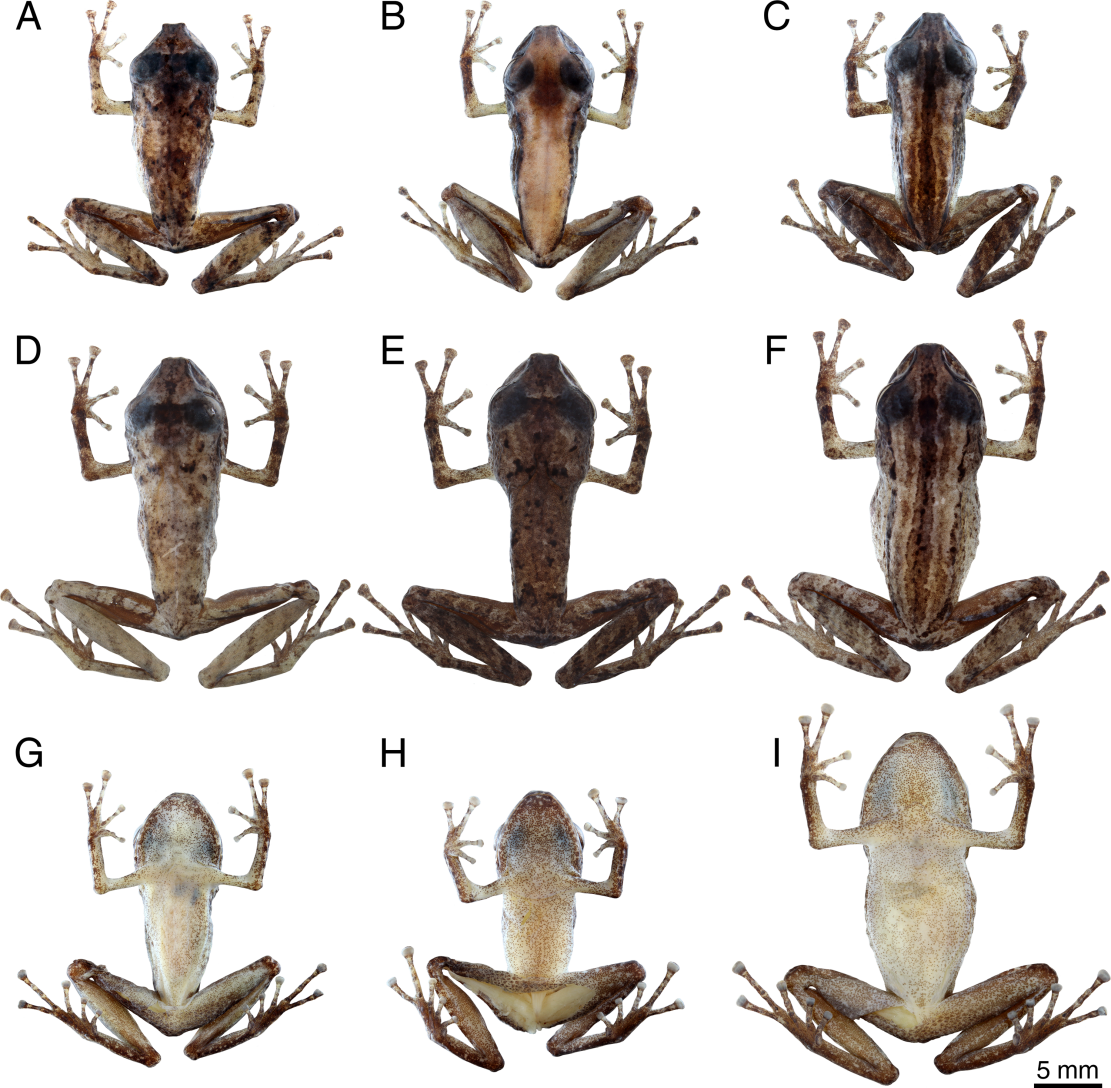
Preserved specimens of *Pristimantiscampinarana* sp. nov. Dorsal view: (A) MPEG 44639, (B) MPEG 44641, (C) INPA-H 44424, (D) INPA-H 44436, (E) INPA-H 44437 and (F) MPEG 44638. Ventral view: (G) MPEG 44641, (H) INPA-H 44424, and (I) MPEG 44638. Males (A–C, G–H). Females (D–F, I). Photographs: A.T. Mônico.

SVL ranges from 17.3 to 20.1 mm in males (*n* = 16) and from 23.2 to 26.5 mm in females (*n* = 6) (Table 1). *Canthus rostralis* almost straight in dorsal view (*e.g*., Fig. 3B, holotype) to slightly curved in some individuals (*e.g*., Figs. 5E and 5F). Three to four ulnar tubercles are present in males (barely visible in 63% of them), ulnar tubercles absent in females. Dorsal skin texture varies from shagreen to granular, small tubercles are present or absent (character of skin texture probably depends on individual activity at the moment of sampling; see Guayasamin et al., 2015; Kok et al., 2018).

In preservative, three types of basic dorsal color patterns can be detected in the type series of *Pristimantis campinarana* sp. nov.: dorsum with irregular dark brown markings (68%; Figs. 5A, 5D and 5E), dorsal coloration sharply outlined against the flanks (9%; Fig. 5B), dorsum with dark brown stripes as in the holotype (23%; Figs. 5C and 5F). In addition, a dark brown interorbital bar is present in 73% of the specimens (Figs. 5A, 5D and 5E); a dark brown W-shaped mark is presented on the scapular region of 18% of the individuals, in some of them more conspicuous (Figs. 5A and 5D) than in others (Figs. 5D and 5E). Dark brown bars and blotches are present on the upper lip of all type specimens, dark and distinct in 73% of the specimens, faded or less conspicuous in the others. Obvious transversal dark brown bars are present on the arms and hands of 63% of the individuals, less conspicuous or absent in 23% and 14% of the specimens, respectively. Distinct transversal dark bars are present on the thigh and tibia of 32% of the specimens (Figs. 5C and 5F), poorly conspicuous in 54% (Fig. 5A) and absent in 14% of the individuals (Fig. 5B). Dark supratympanic stripe present in all specimens. Ventral surface is whitish cream to yellowish white, with a moderate amount of melanophores in 59% of individuals (Fig. 5I), small and large amount in 32% (Fig. 5G) and 9% (Fig. 5H) of individuals, respectively.

**Figure 6:**
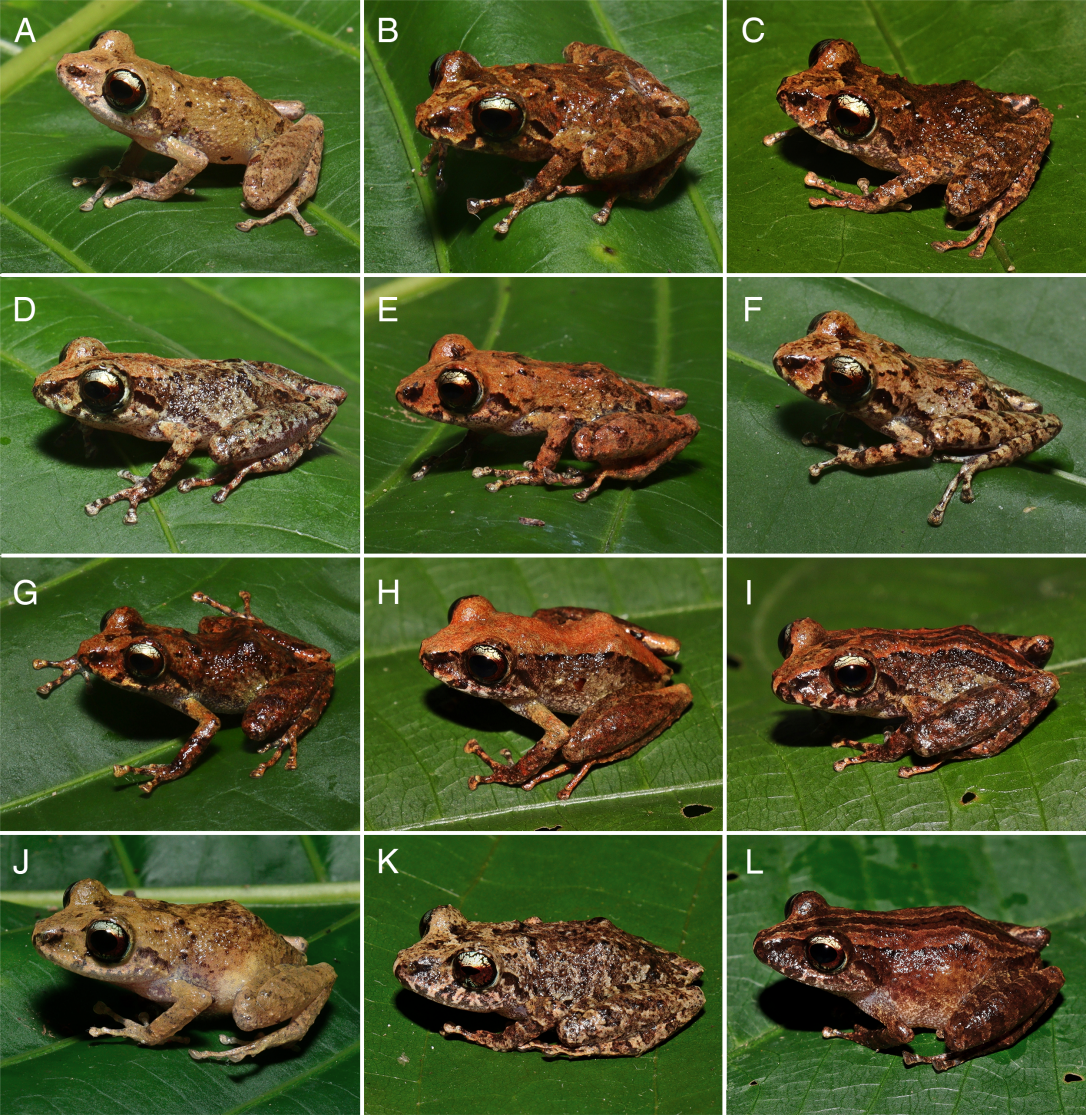
Paratypes of *Pristimantis campinarana* sp. nov. in life. Males: (A) INPA-H 44427 (SVL 19.5 mm), (B) INPA-H 44429 (SVL 18.4 mm), (C) INPA-H 44435 (SVL 18.9 mm), (D) INPA-H 44433 (SVL 18.7 mm), (E) INPA-H 44432 (SVL 18.6 mm), (F) INPA-H 44431 (SVL 19.8 mm), (G) INPA-H 44428 (SVL 17.3 mm), (H) MPEG 44641 (SVL 19.1 mm) and (I) MPEG 44637 (SVL 19.2 mm). Females: (J) INPA-H 44425 (SVL 24.4 mm), (K) INPA-H 44437 (SVL 23.5 mm) and (L) MPEG 44638 (SVL 24.7 mm).

**Figure 7:**
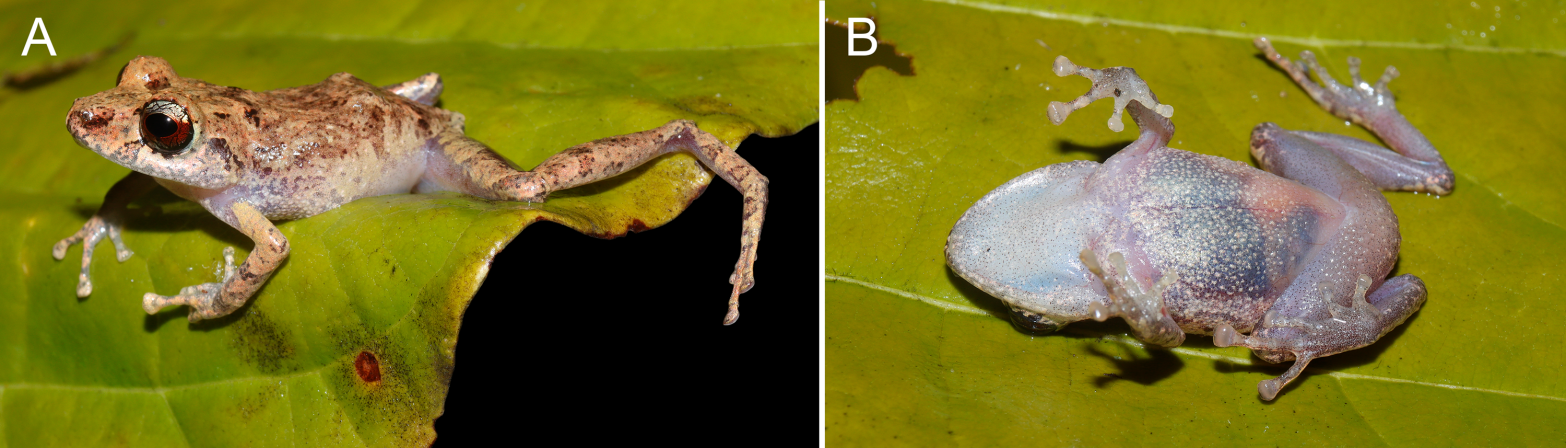
Nocturnal coloration of the groin and ventral surface of *Pristimantis campinarana* sp. nov. in life. (A) Lateral and (B) ventral view of the female (INPA-H 44699, SVL 24.4 mm) at Reserva do Desenvolvimento Sustentável do Rio Negro, municipality of Iranduba, state of Amazonas, Brazil. Photographs: A.T. Mônico.

In life, dorsal background coloration is widely variable, from yellow ocher (color 14 by Köhler, 2012; Figs. 6A, 6D and 6J) and light chrome orange (color 76 by Köhler, 2012; Figs. 6D, 6G and 6J) to antique brown (color 24 by Köhler, 2012; Figs. 6C, 6G, 6I and 6L). Dorsolateral stripe present in some individuals (Figs. 6H and 6I, 6L), but absent in others (Figs. 6A–6G, 6J–6K). Canthal stripe present in some individuals (Figs. 6D, 6H, 6I and 6L), but absent in others (Figs. 6A–6C, 6E–6G, 6J–6K). The iris of most specimens is similar to that of the holotype, but some specimens show a small portion of the lower iris pale bronze to gold (Figs. 6A, 6C, 6I and 6K). At the day, the groin and ventral surface are platt’s-payne’s-gray (color 293 by Köhler, 2012) with brown melanophores (Fig. 5B); at night, when melanophores are less expanded, the groin (Fig. 7A) and ventral surface (Fig. 7B) becomes lighter, almost white.

### Advertisement call

**Figure 8:**
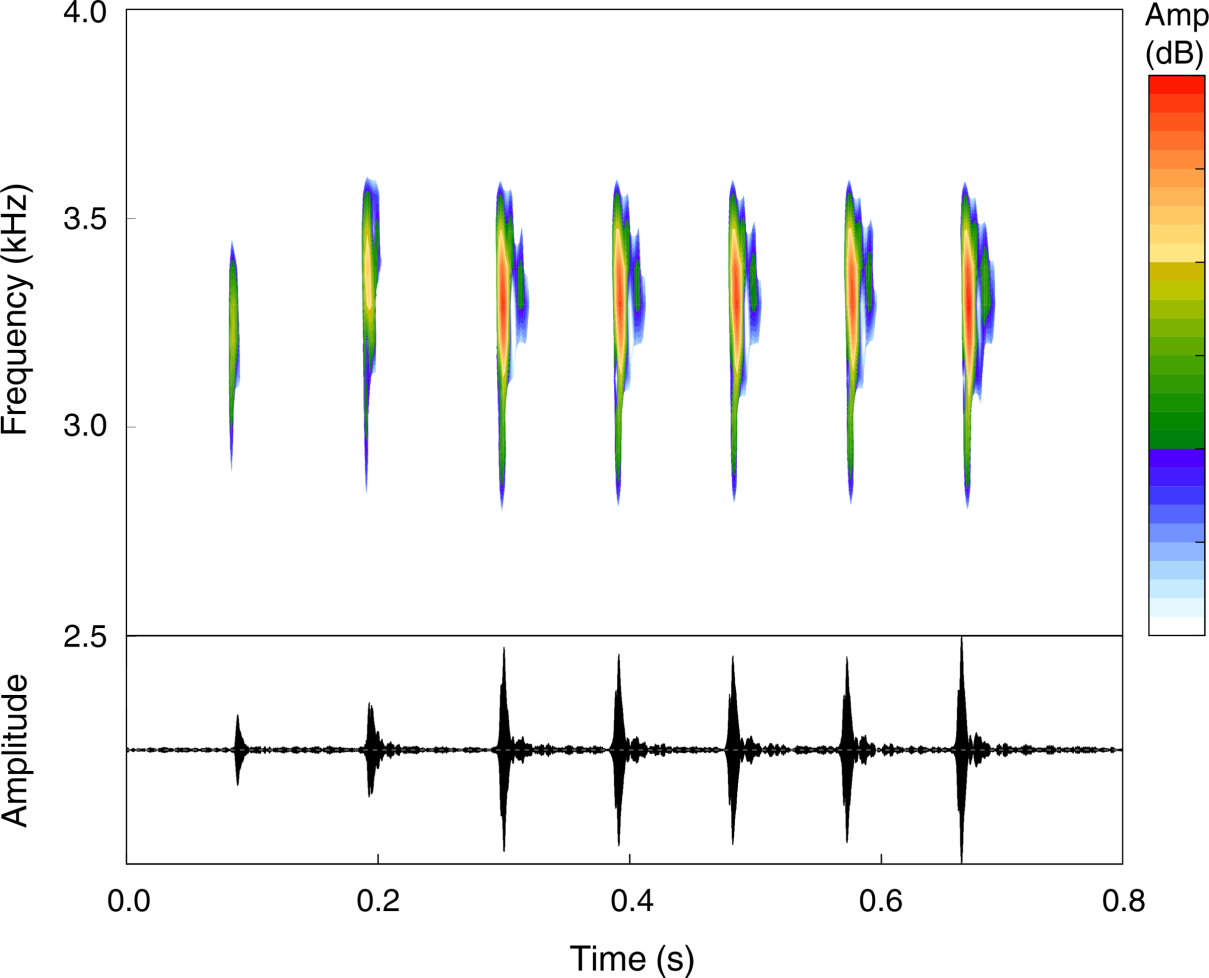
Advertisement call of the holotype (INPA-H 44426, FNJV 59105) of *Pristimantis campinarana* sp. nov. recorded at the Ramal Nova Esperança, municipality of Iranduba, state of Amazonas, Brazil. Air temperature 25.9°C.

The advertisement call of *Pristimantis campinarana* sp. nov. (*n* = 9 males) is composed of 5–10 notes (*n* = 26 calls)—most commonly of 6–8 notes (*n* = 21)—and has a call duration of 694 ± 115 ms (550–1,061 ms). Notes are tonal, have duration of 26.5 ± 7.1 ms (13.2–39.8 ms) and an inter-note interval of 82.7 ± 11.9 ms (64.4–109.4 ms). Calls are emitted with a minimum frequency of 2,853 ± 141 Hz (2,260–3,176 Hz), a maximum frequency of 4,490 ± 413 Hz (3,756–5,280 Hz) and a dominant frequency of 3,587 ± 204 Hz (3,295–3,919 Hz) (Fig. 8). Temporal and spectral traits summarized according to individual call arrangement are presented in Table 2.

### Etymology

The specific epithet ‘*campinarana*’ is used as a noun in apposition and refers to the word in Portuguese that defines the type of forest that the new species occupies: the white-sand forest *campinarana*.

### Distribution, natural history and conservation

**Figure 9:**
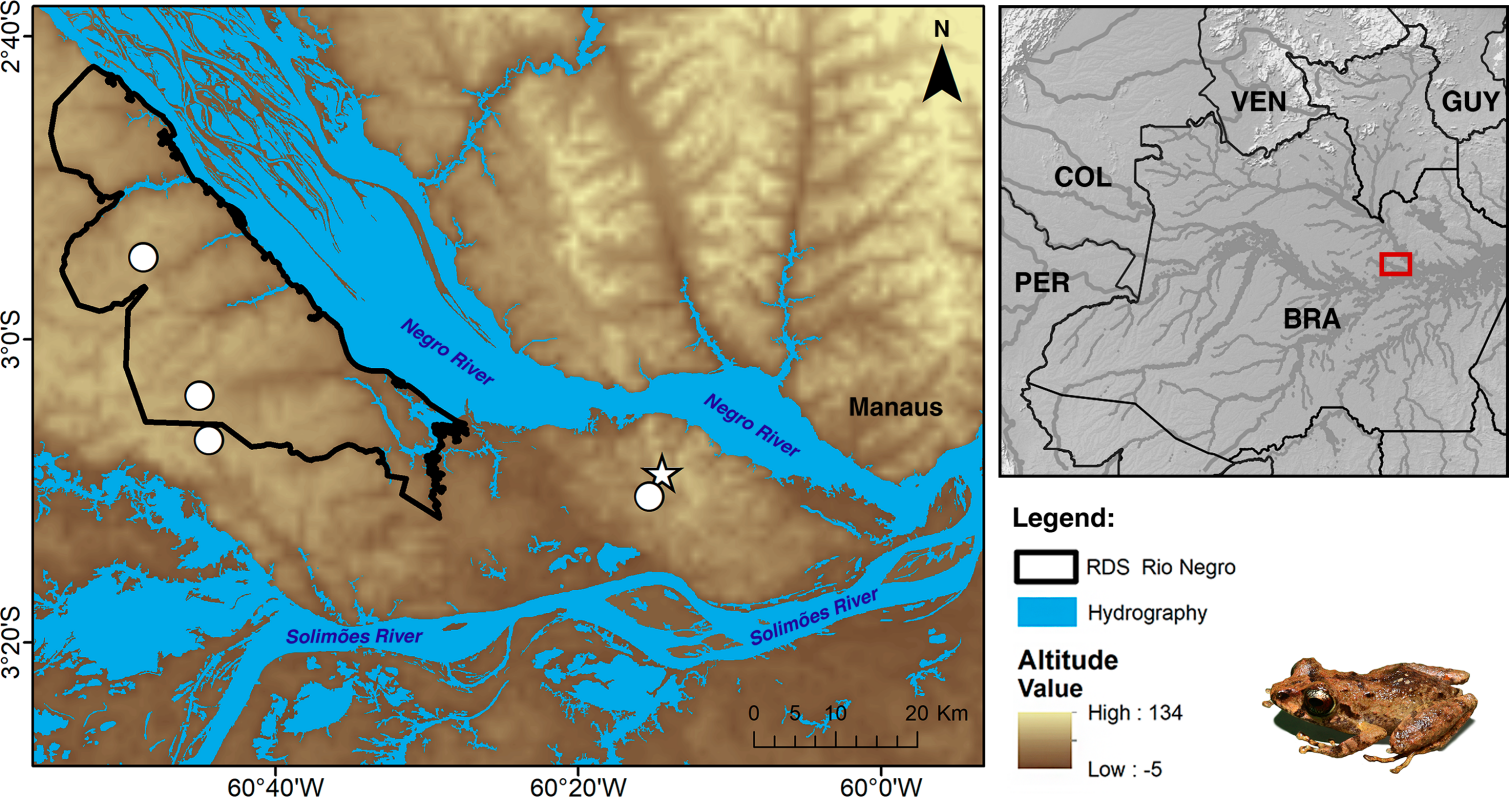
Geographic distribution of *Pristimantis campinarana* sp. nov. Symbols represent the type (star) and paratype (circle) localities. Country acronyms: BRA, Brazil; COL, Colombia; GUY, Guyana; PER, Peru; and VEN, Venezuela.

**Figure 10:**
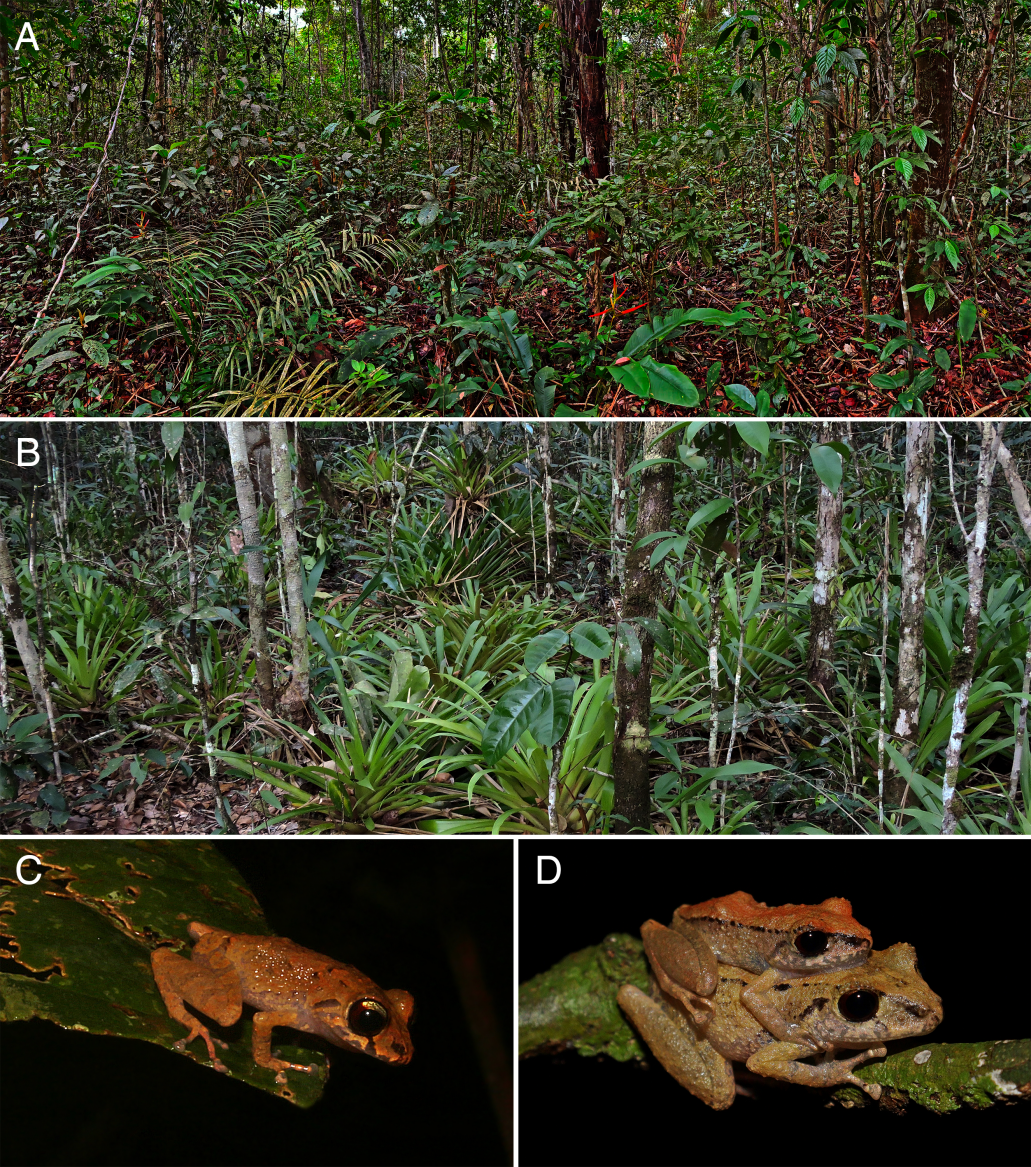
Natural history and breeding aspects of *Pristimantis campinarana* sp. nov. (A) An example of “campinarana” environment inhabited by the new species at Ramal Nova Esperança. (B) Habitat of *P. campinarana* sp. nov. with terrestrial bromeliads at RDS Rio Negro, municipality of Iranduba, Amazonas, Brazil. (C) An unvouchered active calling male perched horizontally on a leave. (D) An amplectant couple (female MPEG 44640, SVL 26.52 mm; male MPEG 44641, SVL 19.15 mm) at Ramal Nova Esperança. Photographs: A.T. Mônico (A, D), J. Moravec (B) and E.D. Koch (C).

Currently, *Pristimantis campinarana* sp. nov. is known only from primary and slightly anthropized forests at two localities in the municipality of Iranduba, state of Amazonas, Brazil (Fig. 9). All individuals were recorded in WSE characterized as *campinarana* (a forest with canopy height below 20 m; Fig. 10A), where the species is locally abundant. However, none was found in *campina*, a white-sand forest with canopy height below 10 m and large patches of exposed white sandy soils.

**Table 1:** Morphometric measurements in millimeters of adult type specimens of *Pristimantis campinarana* sp. nov. Values express mean ± standard deviation, and range.

Morphometric measurements	*Pristimantis campinarana* sp. nov.		
	Holotype	Males^*^ (*n* = 16)	Females (*n* = 6)
SVL	19.1	18.9 ± 0.7 (17.3–20.1)	24.5 ± 1.2 (23.2–26.5)
HW	6.8	6.7 ± 0.3 (6.1–7.0)	8.8 ± 0.6 (8.0–9.8)
HL	7.0	7.1 ± 0.2 (6.6–7.3)	9.2 ± 0.5 (8.5–10.1)
SL	2.9	2.8 ± 0.1 (2.6–3.1)	3.6 ± 0.2 (3.2–3.9)
IND	1.9	1.8 ± 0.1 (1.7–1.9)	2.3 ± 0.2 (2.0–2.6)
EN	2.2	2.2 ± 0.1 (2.0–2.4)	2.8 ± 0.3 (2.7–3.2)
IOD	2.2	2.3 ± 0.1 (2.1–2.4)	2.9 ± 0.1 (2.7–3.1)
ED	2.7	2.8 ± 0.1 (2.6–3.0)	3.2 ± 0.1 (3.0–3.3)
TD	0.9	0.9 ± 0.1 (0.8–1.0)	1.2 ± 0.1 (1.2–1.4)
UAL	5.2	5.0 ± 0.3 (4.4–5.4)	6.7 ± 0.3 (6.3–7.0)
FAL	4.3	4.2 ± 0.2 (3.8–4.7)	5.8 ± 0.4 (5.4–6.3)
HAND	5.2	4.8 ± 0.2 (4.6–5.3)	6.4 ± 0.4 (6.0–7.3)
HANDI	2.6	2.5 ± 0.2 (2.2–2.8)	3.4 ± 0.3 (3.2–3.9)
HANDII	3.4	3.3 ± 0.2 (3.0–3.6)	4.2 ± 0.4 (3.8–4.9)
HANDIV	4.2	4.1 ± 0.2 (3.7–4.3)	5.3 ± 0.4 (4.9–6.2)
WFD	0.8	0.8 ± 0.1 (0.7–1.0)	1.1 ± 0.1 (0.9–1.2)
THL	9.4	9.4 ± 0.2 (9.1–9.7)	11.7 ± 0.8 (11.2–13.2)
TL	9.7	9.8 ± 0.3 (9.3–10.4)	12.5 ± 0.4 (11.8–13.7)
TAL	4.9	4.9 ± 0.3 (4.6–5.5)	6.4 ± 0.4 (5.9–7.1)
FL	7.9	7.8 ± 0.3 (7.4–8.3)	10.0 ± 0.7 (9.5–11.1)
FLI	2.2	2.3 ± 0.2 (2.1–2.8)	3.2 ± 0.2 (3.0–3.6)
FLII	3.4	3.3 ± 0.2 (3.1–3.7)	4.5 ± 0.3 (4.2–4.9)
FLIII	5.5	5.2 ± 0.3 (4.7–5.6)	6.8 ± 0.5 (6.4–7.6)
FLV	6.4	6.3 ± 0.3 (6.0–7.1)	8.2 ± 0.6 (7.6–9.2)
WTD	0.9	0.8 ± 0.1 (0.7–1.0)	1.2 ± 0.1 (1.0–1.4)

**Notes.**

* It includes the holotype’s measurements.

**Table 2:** Acoustic traits of the call of *Pristimantis campinarana* sp. nov. summarized according to call arrangements. Temporal and spectral traits are presented in milliseconds and Hz, respectively.

Call arrangement		CD	ND	INI	LF	HF	DF
5 notes (*n* = 2)	mean	605	35	92	2,941	4,546	3,553
	SD	12	5	7	332	2.8	274
	min	596	31	98	2,706	4,544	3,359
	max	613	38	87	3,176	4,548	3,747
6 notes (*n* = 9)	mean	624	24	87	2,888	4,528	3,637
	SD	47	9	12	139	503	267
	min	550	13	72	2,682	3,785	3,316
	max	691	39	109	3,064	5,241	3,919
7 notes (*n* = 8)	mean	679	30	77	2,778	4,321	3,486
	SD	57	5	6	95	375	176
	min	601	23	69	2,686	3,756	3,295
	max	748	40	84	2,947	4,773	3,768
8 notes (*n* = 5)	mean	749	24	79	2,832	4,397	3,630
	SD	25	2	6	126	66	78
	min	710	22	70	2,660	4,287	3,531
	max	779	27	86	2,977	4,456	3,725
9 notes (*n* = 1)	–	974	23	95	2,904	5,053	3,725
10 notes (*n* = 1)	–	1,065	21	95	3,019	5,279	3,661

**Notes.**

SD standard deviation CD call duration ND note duration NN number of notes per call INI inter-note interval LF minimum frequency HF maximum frequency DF dominant frequency

*Pristimantis campinarana* sp. nov. is a crepuscular and nocturnal species, with peak activity at crepuscule. Its breeding activity takes place in the rainy season (November to February). In the dry season, we found males sheltering among the leaves of terrestrial bromeliads of the genus *Guzmania* (Fig. 10B). In the rainy season, males start calling at dusk (∼18:00 h) and are very active until ∼20:00 h. Then their activity decrease and end usually around 22:00 h. In rainy days, the vocalization is sustained throughout the night. Males were observed calling perched on the vegetation (Fig. 10C) usually 1 m above the ground, rarely above 3 m. Calling males aggregate in groups of up to ten individuals, separated from each other by ∼4–5 m, but it is not uncommon to observe smaller groups of three to four calling individuals that are spatially more spaced. The amplexus (*n* = 3) is axillary (Fig. 10D). Clutches were not found *in situ*, but females produce about 14–17 large oocytes (*n* = 4). The new species occurs in sympatry with a candidate species closely related to *Pristimantis orcus* (AT Mônico, 2022, unpublished data). We do not have sufficient data to categorize the new species following the criteria of the International Union for Conservation of Nature (IUCN), it should be thus considered Data Deficient (DD).

## Discussion

The new species described herein is currently known only from white-sand forests from the Negro-Solimões interfluve. In Amazonia most of WSE is found within this interfluve, peppered within a matrix of dense forest (Adeney et al., 2016). Although long-term herpetological surveys were conducted in dense ombrophilous forests east of Negro River and in the Purus-Madeira Interfluve, the new species was never found there. Therefore, we assume that the new species is endemic of the Negro-Solimões interfluve and only found in WSE. This distributional pattern is possibly similar to the ones of other anuran species (*Trachycephalus venezolanus* [Mertens, 1950], *Osteocephalus vilarsi* [Melin, 1941] and *Scinax albertinae* Ferrão et al., 2022) recently discovered from the Negro-Solimões interfluve (Ferrão et al., 2019; Ferrão et al., 2022). Moreover, additional undescribed species (*e.g*., species of *Adenomera*; M Ferrão, 2022, unpublished data; *Phyllomedusa* AP Lima, 2022, unpublished data; *Pristimantis* aff. *orcus*, AT Mônico, 2022, unpublished data) are also found associated to WSE, thus totaling at least seven frog species sharing this habitat specialization and distribution pattern endemic to the Negro-Solimões interfluve. In fact, the Jaú region was recently defined as an additional independent area of endemism in Amazonia due to the coocurrence of six bird species (Borges & Da Silva, 2012). Our finding, thus, strengthen the idea that white-sand ecosystems of the Negro-Solimões interfluve harbors a unique biodiversity that deserves effective protection.

*Pristimantis campinarana* is nested within a clade formed by species otherwise restricted to western Amazonia lowlands: *P. matidiktyo*, *P. ockendeni*, *P. delius* and *P. librarius.* This nested position of *P. campinarana* suggests that the speciation occurred after a dispersal from the west. A possible scenario involves historical changes in the Amazon basin drainage (Hoorn et al., 2010; Albert, Val & Hoorn, 2018) such as the disappearance of a riverine barrier that connected the Japurá River to Negro River (Ruokolainen et al., 2019), in the Jaú region, that could have favored the dispersal *P. campinarana* ancestors. Relatively recent eastward dispersals have been reported for other anuran groups, from small leaflitter toads (*i.e., Allobates caeruleodactylus* and *A. trilineatus* clades, Réjaud et al., 2020; *Ameerega*, Roberts et al., 2006) to arboreal treefrogs(*i.e., Osteocephalus taurinus* and *O. buckleyi* groups, Ortiz et al., 2022). Alternative scenarios involve dispersal across rivers (Smith et al., 2014; Moraes et al., 2016; Pirani et al., 2019). Both hypotheses, however, involve subsequent isolation by river and eventually speciation (Wallace, 1852; Haffer, 1974; *e.g.*, Ribas et al., 2012; Rojas et al., 2018).

Despite large genetic divergence and consistent diagnosis characters, the species forming this clade remain very similar in morphology (*e.g.*, SVL of males, ventral skin texture and iris in life) illustrating the trend in the genus of highly conserved morphology and the challenge that describing its diversity (Padial & De la Riva, 2009; Padial et al., 2009; Waddell et al., 2018; De Oliveira et al., 2020). This phenotypic conservatism have been discussed for other Amazonian frogs (Gonzalez-Voyer et al., 2011; Kaefer et al., 2013; Guayasamin et al., 2015; De Oliveira et al., 2020), as well as its implications for taxonomy (Funk et al., 2007; Kaefer et al., 2013; Waddell et al., 2018).

New species are being described recurrently in Amazonia, even from areas close to large urban and research centers. For example, in the last years, several new species were described from the Reserva Florestal Adolpho Ducke (Manaus, Brazil), an intensively studied area (*e.g.*, *Amazophrynella manaos* Rojas-Zamora et al., 2014; *Atelopus manauensis* Jorge, Ferrão & Lima, 2020; *Synapturanus ajuricaba* Fouquet et al., 2021; and *Pristimantis guianensis* Mônico et al., 2022). The lowlands in the Negro-Solimões interfluve are also close to Manaus, and also harbor newly described species notably the discovery of a new lizard genus (*i.e*., *Marinussaurus* Peloso et al., 2011). This illustrates how far we are from understanding the species diversity in Amazonia particularly for small and cryptic species like the *P. unistrigatus* group (Fouquet et al., 2022a; Mônico et al., 2022).

## Conclusions

Using morphology, bioacoustics and molecular data from three markers (16S, COI and RAG-1), we described a novel species of rain frog (genus *Pristimantis*) from an unexplored environment in the Amazonia: the white sand ecosystems. Description of *Pristimantis campinarana* sp. nov. reaffirms that the species richness of the west Amazonian anurans remains considerably underestimated even near to the largest and dynamically developing Amazonian metropole—Manaus. The congruence of seven frog species sharing a WSE specialization and distribution pattern likely endemic to the Negro-Solimões interfluve seem to reinforce the area of endemism proposed by Borges & Da Silva (2012) in the Jaú region. Also, the vertebrate fauna of white sand forests is unique and recent studies have revealed notable discoveries, reinforcing the need for effective protection of these environments.

##  Supplemental Information

10.7717/peerj.15399/supp-1 Table S1 Morphometric measurements (in mm) of adults of the type series of *Pristimantis campinarana* sp. nov Measurement acronyms are defined in the text. Abbreviations: INPAH, Instituto Nacional de Pesquisas da Amazônia; MPEG, Museu Paraense Emílio Goeldi; FN, field numbers; M, male; F, female.

10.7717/peerj.15399/supp-2 Table S2 Acoustic parameters of advertisement call of *Pristimantis campinarana* sp. nov Abbreviations: vouchers: INPAH, Instituto Nacional de Pesquisas da Amazônia; MPEG, Museu Paraense Emílio Goeldi; FNJV, Fonoteca Neotropical Jacques Vielliard; AT, air temperature (ºC); NN, number of notes per call; CD, call duration (ms); ND, note duration (ms); INI, inter-note interval (ms); LF, minimum frequency (Hz); HF, maximum frequency (Hz); and DF, dominant frequency (Hz).

## Figures and Tables

**Appendix 2:**
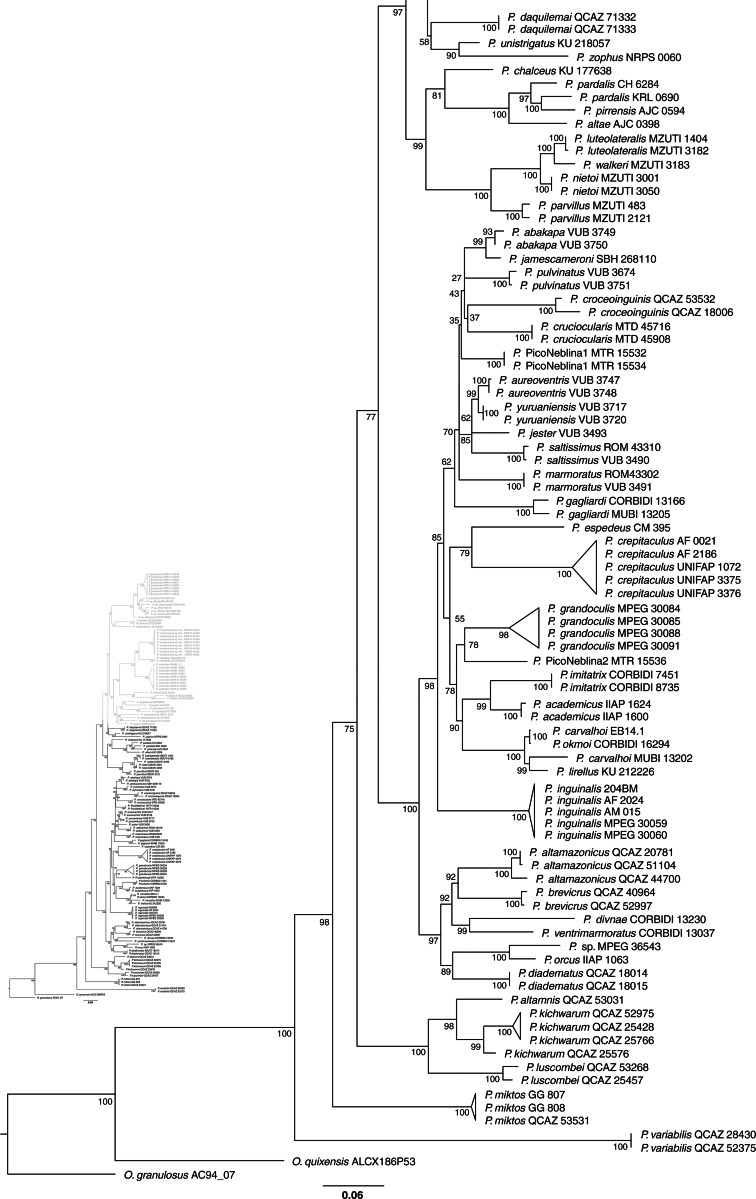
Phylogenetic reconstruction showing the position of *Pristimantis campinarana* sp. nov. within the *P. unistrigatus* species group. Maximum likelihood tree inferred with 16S, COI and RAG1. Non-parametric ultrafast bootstrap support is shown close to nodes.

## Appendix 1

**Appendix 1:** Specimens of *Pristimantis* and *Oreobates* used in phylogenetic analyses, with respective voucher, GenBank acession number and references.

**Species**	**Voucher**	**GenBank acession numbers**			**Reference**
		**16S**	**COI**	**RAG1**	
*P. abakapa*	VUB3749	JQ742162			Kok et al. (2012)
*P. abakapa*	VUB3750	JQ742163			Kok et al. (2012)
*P. academicus*	IIAP1624	OL989867	OL960436		Ortega-Andrade, Deichmann & Chaparro (2021)
*P. academicus*	IIAP1600	OL989866			Ortega-Andrade, Deichmann & Chaparro (2021)
*P. altae*	AJC0398		JN991361	JQ025174	Pinto-Sanchez et al. (2012)
*P. ardalonychus*	KU212301	EU186664			Hedges, Duellman & Heinicke (2008)
*P. altamazonicus*	QCAZ44700	MF118685	MF118717	MF118735	Ortega-Andrade et al. (2017)
*P. altamazonicus*	QCAZ20781	MF118699	MF118707	MF118734	Ortega-Andrade et al. (2017)
*P. altamazonicus*	QCAZ51104	MF118678	MF118720	MF118738	Ortega-Andrade et al. (2017)
*P. altamnis*	QCAZ53031	KP064155	KP064164		Ortega-Andrade & Venegas (2014)
*P. aureoventris*	VUB3747	JQ742154			Kok et al. (2012)
*P. aureoventris*	VUB3748	JQ742152			Kok et al. (2012)
*P. bogotensis*	NRPS0033	JN991432	JN991362		Pinto-Sanchez et al. (2012)
*P. brevicrus*	QCAZ52997	MF118697	MF118726	MF118744	Ortega-Andrade et al. (2017)
*P. brevicrus*	QCAZ40964	MF118700	MF118715	MF118751	Ortega-Andrade et al. (2017)
*P. buenaventura*	MZUTI3270	KU999169			Arteaga et al. (2016)
*P. cajamarcensis*	KU217845	EF493663			Heinicke, Duellman & Hedges (2007)
*P. campinarana* sp. nov.	MPEG44634	OP965363	OP964730	OP980951	This study
*P. campinarana* sp. nov.	MPEG44635	OP965364	OP964731	OP980952	This study
*P. campinarana* sp. nov.	INPA-H44439	OP965362	OP964732	OP980953	This study
*P. campinarana* sp. nov.	INPA-H44424	OP965356	OP964733	OP980954	This study
*P. campinarana* sp. nov.	INPA-H44426	OP965357	OP964734	OP980955	This study
*P. campinarana* sp. nov.	INPA-H44427	OP965358	OP964735	OP980956	This study
*P. campinarana* sp. nov.	INPA-H44428	OP965359	OP964736	OP980957	This study
*P. campinarana* sp. nov.	INPA-H44429	OP965360	OP964737	OP980958	This study
*P. campinarana* sp. nov.	INPA-H44431	OP965361	OP964738	OP980959	This study
*P. carvalhoi*	MUBI13202	OL989869			Ortega-Andrade, Deichmann & Chaparro (2021)
*P. carvalhoi*	EB14.13	MG820152	MG820177		Catenazzi & Lehr (2018)
*P. ceuthospilus*	KU212216	EF493520			Heinicke, Duellman & Hedges (2007)
*P. chalceus*	KU177638	EF493675			Heinicke, Duellman & Hedges (2007)
*P. crepitaculus*	AF2786	KDQF01001103			Fouquet et al. (2022a)
*P. crepitaculus*	21AF	JN691315			Fouquet et al. (2022a)
*P. crepitaculus*	UNIFAP 1072	ON908889	ON954784	ON963983	Mônico et al. (2022)
*P. crepitaculus*	UNIFAP 3375	ON908890	ON954785	ON963984	Mônico et al. (2022)
*P. crepitaculus*	UNIFAP 3376	ON908891	ON954786	ON963985	Mônico et al. (2022)
*P. croceoinguinis*	QCAZ53532	KP064144	KP064157	MF118752	Ortega-Andrade & Venegas (2014)
*P. croceoinguinis*	QCAZ18006	MH516170			Waddell et al. (2018)
*P. cruciocularis*	MTD45716	MG820161	MG820186		Catenazzi & Lehr (2018)
*P. cruciocularis*	MTD45908	MG820162	MG820187		Catenazzi & Lehr (2018)
*P. daquilemai*	QCAZ71332	MZ430056			Brito-Zapata et al. (2021)
*P. daquilemai*	QCAZ71333	MZ430057			Brito-Zapata et al. (2021)
*P. delius*	QCAZ53035	KP064150	KP064162	MF118753	Ortega-Andrade & Venegas (2014)
*P. diadematus*	QCAZ18014	MH516177			Waddell et al. (2018)
*P. diadematus*	QCAZ18015	MH516178			Waddell et al. (2018)
*P. divnae*	CORBIDI13230	OL989858	OL960443		Ortega-Andrade, Deichmann & Chaparro (2021)
*P. espedeus*	CM395	JN691314			Fouquet et al. (2013)
*P. gagliardi*	CORBIDI13166	OL989857	OL960444		Ortega-Andrade, Deichmann & Chaparro (2021)
*P. gagliardi*	MUBI13205	OL989868	OL960435		Ortega-Andrade, Deichmann & Chaparro (2021)
*P. grandoculis*	MPEG 30085	KDQF01002880			Fouquet et al. (2022a)
*P. grandoculis*	MPEG 30088	KDQF01002881			Fouquet et al. (2022a)
*P. grandoculis*	MPEG 30084	ON908894	ON964531	ON963988	Mônico et al. (2022)
*P. grandoculis*	MPEG 30091	ON908895	ON964532	ON963989	Mônico et al. (2022)
*P. guianensis*	INPA-H 43918	ON897772	ON898573	ON920937	Mônico et al. (2022)
*P. guianensis*	INPA-H 43919	ON897773	ON898574	ON920938	Mônico et al. (2022)
*P. guianensis*	INPA-H 43920	ON897774	ON898575	ON920939	Mônico et al. (2022)
*P. guianensis*	INPA-H 43921	ON897775	ON898576	ON920940	Mônico et al. (2022)
*P. guianensis*	INPA-H 43923	ON897776	ON898577	ON920941	Mônico et al. (2022)
*P. guianensis*	INPA-H 43924	ON897777	ON898578	ON920942	Mônico et al. (2022)
*P. guianensis*	INPA-H 43925	ON897778	ON898579	ON920943	Mônico et al. (2022)
*P. guianensis*	INPA-H 43943	ON897779	ON898580	ON920944	Mônico et al. (2022)
*P. imitatrix*	CORBIDI7451	OL989854			Ortega-Andrade, Deichmann & Chaparro (2021)
*P. imitatrix*	CORBIDI8735	OL989855			Ortega-Andrade, Deichmann & Chaparro (2021)
*P. inguinalis*	204BM	JN691317			Fouquet et al. (2013)
*P. inguinalis*	AM015	KDQF01001691			Vacher et al. (2020)
*P. inguinalis*	AF2054	KDQF01000814			Vacher et al. (2020)
*P. inguinalis*	MPEG 30059	ON908892	ON964824	ON963986	Mônico et al. (2022)
*P. inguinalis*	MPEG 30060	ON908893	ON964825	ON963987	Mônico et al. (2022)
*P. jamescameroni*	SBH268110	EU186721			Hedges, Duellman & Heinicke (2008)
*P. jester*	VUB3493	JQ742169			Kok et al. (2012)
*P. kichwarum*	QCAZ52975	KP064154			Ortega-Andrade & Venegas (2014)
*P. librarius*	QCAZ25852	JN991451	JN991379	JQ025188	Pinto-Sanchez et al. (2012)
*P. librarius*	QCAZ25853	MH516184		MH481370	Waddell et al. (2018)
*P. lirellus*	KU212226	EF493521			Heinicke, Duellman & Hedges (2007)
*P. luscombei*	QCAZ53268	KP064156			Ortega-Andrade & Venegas (2014)
*P. luscombei*	QCAZ25457	EU130618		MH481368	Elmer, Dávila & Lougheed (2007)
*P. luteolateralis*	MZUTI3182	KU999208			Arteaga et al. (2016)
*P. luteolateralis*	MZUTI1404	KU999205			Arteaga et al. (2016)
*P. marmoratus*	ROM43302	EU186716			Kok et al. (2018)
*P. marmoratus*	VUB3491	JQ742167			Kok et al. (2018)
*P. martiae*	QCAZ52983	KP064148	KP064160	MF118754	Ortega-Andrade & Venegas (2014)
*P. martiae*	QCAZ52984	KP064149	KP064161	MF118755	Ortega-Andrade & Venegas (2014)
*P. matidiktyo*	QCAZ 53021	KP064147	KP064159		Ortega-Andrade & Venegas (2014)
*P. matidiktyo*	QCAZ 52779	KP064146	KP064158		Ortega-Andrade & Venegas (2014)
*P. miktos*	QCAZ 53531	KP064153			Ortega-Andrade & Venegas (2014)
*P. miktos*	GGU807	KP064151			Ortega-Andrade & Venegas (2014)
*P. miktos*	GGU808	KP064152	KP064163		Ortega-Andrade & Venegas (2014)
*P. miyatai*	AJC3475	KP149490	KP149276		Guarnizo et al. (2015)
*P. nietoi*	MZUTI3050	KU999214			Arteaga et al. (2016)
*P. nietoi*	MZUTI3001	KU999212			Arteaga et al. (2016)
*P. ockendeni*	RvM5_12	KY652654	KY672986	KY672970	Von May et al. (2017)
*P. ockendeni*	INPA-H 43945	ON897793	ON898594	ON920954	Mônico et al. (2022)
*P. ockendeni*	INPA-H 43946	ON897794	ON898595	ON920955	Mônico et al. (2022)
*P. ockendeni*	INPA-H 43947	ON897795	ON898596	ON920956	Mônico et al. (2022)
*P. ockendeni*	INPA-H 43949	ON897796	ON898597	ON920957	Mônico et al. (2022)
*P. ockendeni*	INPA-H 43952	ON897797	ON898598	ON920958	Mônico et al. (2022)
*P. ockendeni*	MUBI 10538	ON907779			Mônico et al. (2022)
*P. ockendeni*	MUBI 13049	ON907778			Mônico et al. (2022)
*P. ockendeni*	MUBI 14568	ON907780			Mônico et al. (2022)
*P. orcus*	IIAP1063	OL989865	OL960437		Ortega-Andrade, Deichmann & Chaparro (2021)
*P. okmoi*	CORBIDI16294	KY652651	KY672983	KY672967	Ortega-Andrade, Deichmann & Chaparro (2021)
*P. pardalis*	KRL0690	FJ784336	FJ766804	JQ025198	Crawford, Lips & Bermingham (2010)
*P. pardalis*	CH6284	JN991460	JN991390		Pinto-Sanchez et al. (2012)
*P. parvillus*	MZUTI483	KU999215			Arteaga et al. (2016)
*P. parvillus*	MZUTI2121	KU999216			Arteaga et al. (2016)
*P. pirrensis*	AJC0594	JN991462	JN991393	JQ025199	Pinto-Sanchez et al. (2012)
*P. pulvinatus*	VUB3751	JQ742164			Kok et al. (2018)
*P. pulvinatus*	VUB3674	JQ742165			Kok et al. (2018)
*P. saltissimus*	VUB3490	JQ742168			Kok et al. (2012)
*P. saltissimus*	ROM43310	EU186693			Hedges, Duellman & Heinicke (2008)
*P.* sp. PicoNeblina1	MTR15532	KDQF01003322			Vacher et al. (2020)
*P.* sp. PicoNeblina1	MTR15534	KDQF01003323			Vacher et al. (2020)
*P.* sp. PicoNeblina2	MTR15536	KDQF01003323			Vacher et al. (2020)
*P.* sp.	MUBI 17035	ON907781			Mônico et al. (2022)
*P.* sp.	MTR 12855	KDQF01003224			Fouquet et al. (2022a)
*P.* sp.	JOG 847	KDQF01002705			Fouquet et al. (2022a)
*P.* sp.	BM 153	KDQF01001829			Fouquet et al. (2022a)
*P.* sp.	MTR 12690	KDQF01003210			Fouquet et al. (2022a)
*P.* sp.	H2773	KDQF01002605			Fouquet et al. (2022a)
*P.* sp.	SMS155	KDQF01004256			Fouquet et al. (2022a)
*P.* sp.	SMS156	KDQF01004257			Fouquet et al. (2022a)
*P. taeniatus*	AJC1839	JN991470	JN991403	JQ025208	Pinto-Sanchez et al. (2012)
*P. taeniatus*	AJC1126	JN991472	JN991406	JQ025206	Pinto-Sanchez et al. (2012)
*P. stictogaster*	KU291659	EF493704		EF493445	Heinicke, Duellman & Hedges (2007)
*P. unistrigatus*	KU218057	EF493387		EF493444	Heinicke, Duellman & Hedges (2007)
*P. variabilis*	QCAZ52375	MT372717		MT372609	Reyes-Puig et al. (2020)
*P. variabilis*	QCAZ28430	MH516198			Waddell et al. (2018)
*P. ventrimarmoratus*	CORBIDI13037	OL989856	OL960445		Ortega-Andrade, Deichmann & Chaparro (2021)
*P. walkeri*	MZUTI3183	KU999230			Arteaga et al. (2016)
*P. yuruaniensis*	VUB3717	JQ742160			Kok et al. (2012)
*P. yuruaniensis*	VUB3720	JQ742161			Kok et al. (2012)
*P. zophus*	NRPS0060	JN991479	JN991414	JQ025213	Pinto-Sanchez et al. (2012)
*P. zophus*	NRPS0072	JN991478	JN991413	JQ025214	Pinto-Sanchez et al. (2012)
*O. quixensis*	ALCX186P53	KU495404	KU494611		Lyra, Haddad & Azeredo-Espin (2016)
*O. granulosus*	AC94_07	KY652649	KY672982	KY672965	Von May et al. (2017)

## Additional Information and Declarations

### DNA Deposition

The following information was supplied regarding the deposition of DNA sequences: The sequences are available at GenBank: OP965356–OP965364 (16S), OP964730–OP964738 (COI) and OP980951–OP980959 (RAG-1).

### Data Availability

The following information was supplied regarding data availability: The raw morphometric and bioacoustic data of adults of the new species are available in the Supplemental Files.

### New Species Registration

The following information was supplied regarding the registration of a newly described species: Publication LSID: urn:lsid:zoobank.org:pub:F8ED54F6-9E18-49BD-8178-659BDFB79C65 Pristimantis campinarana LSID: urn:lsid:zoobank.org:act:61E81E96-5309-4FA0-AE8A-B34636CD5616
