# Supplementary material for: A new species of Pristimantis (Anura: Strabomantidae) from white-sand forests of central Amazonia, Brazil
Source: PeerJ. 2023 Jun 6;11:e15399. doi: 10.7717/peerj.15399 (PMC10252896; doi:10.7717/peerj.15399)
Supplement: Table S1 — Measurement acronyms are defined in the text. Abbreviations: INPAH, Instituto Nacional de Pesquisas da Amazônia; MPEG, Museu Paraense Emílio Goeldi; FN, field numbers; M, male; F, female. [file peerj-11-15399-s001.docx]

| Voucher |  | Sex | SVL | HW | HL | SL | IND | EN | IOD | ED | TD | UAL | FAL | HAND | HANDI | HANDII | HANDIV | WFD | THL | TL | TAL | FL | FLI | FLII | FLIII | TV | WTD |
| --- | --- | --- | --- | --- | --- | --- | --- | --- | --- | --- | --- | --- | --- | --- | --- | --- | --- | --- | --- | --- | --- | --- | --- | --- | --- | --- | --- |
| INPAH 44424 | APL 23162 | M | 18.88 | 6.82 | 7.05 | 2.81 | 1.69 | 2.31 | 2.44 | 2.81 | 0.98 | 5.36 | 4.05 | 4.92 | 2.36 | 3.25 | 3.69 | 0.88 | 9.70 | 9.89 | 4.57 | 7.92 | 2.51 | 3.53 | 5.40 | 6.31 | 0.98 |
| INPAH 44425 | APL 23163 | F | 24.15 | 8.73 | 8.83 | 3.77 | 2.31 | 3.12 | 2.98 | 3.29 | 1.20 | 6.82 | 5.50 | 6.18 | 3.21 | 3.81 | 5.07 | 0.87 | 11.33 | 11.82 | 5.91 | 9.48 | 2.99 | 4.30 | 6.52 | 8.12 | 1.38 |
| INPAH 44426 | APL 23164 | M | 19.05 | 6.84 | 7.03 | 2.87 | 1.93 | 2.20 | 2.20 | 2.72 | 0.99 | 5.17 | 4.25 | 5.23 | 2.62 | 3.41 | 4.18 | 0.84 | 9.40 | 9.72 | 4.91 | 7.88 | 2.20 | 3.44 | 5.50 | 6.44 | 0.93 |
| INPAH 44427 | APL 23165 | M | 19.55 | 7.00 | 7.20 | 2.99 | 1.91 | 2.37 | 2.30 | 2.65 | 1.02 | 4.97 | 4.32 | 5.25 | 2.55 | 3.37 | 4.33 | 0.84 | 9.70 | 10.27 | 5.44 | 8.25 | 2.33 | 3.48 | 5.36 | 6.64 | 0.89 |
| INPAH 44428 | APL 23166 | M | 17.28 | 6.05 | 6.62 | 2.63 | 1.74 | 2.02 | 2.09 | 2.67 | 0.77 | 4.41 | 3.79 | 4.69 | 2.15 | 3.01 | 3.83 | 0.70 | 9.19 | 9.47 | 4.72 | 7.36 | 2.23 | 3.09 | 4.76 | 6.11 | 0.73 |
| INPAH 44429 | APL 23167 | M | 18.42 | 6.81 | 7.10 | 2.77 | 1.84 | 2.31 | 2.29 | 2.72 | 0.69 | 5.08 | 4.37 | 4.65 | 2.39 | 3.08 | 3.99 | 0.83 | 9.07 | 9.56 | 4.86 | 7.50 | 2.08 | 3.06 | 4.93 | 6.03 | 0.82 |
| INPAH 44431 | APL 23169 | M | 19.80 | 6.90 | 7.32 | 3.05 | 1.78 | 2.33 | 2.22 | 2.81 | 1.02 | 5.09 | 4.10 | 4.71 | 2.24 | 3.06 | 3.78 | 0.79 | 9.33 | 9.56 | 4.61 | 7.41 | 2.19 | 3.09 | 4.91 | 5.98 | 0.80 |
| INPAH 44432 | APL 23170 | M | 18.58 | 6.87 | 7.00 | 2.96 | 1.86 | 2.36 | 2.21 | 2.94 | 0.92 | 5.10 | 4.14 | 5.08 | 2.67 | 3.34 | 4.17 | 0.97 | 9.43 | 9.83 | 4.70 | 7.73 | 2.50 | 3.45 | 5.07 | 6.18 | 0.86 |
| INPAH 44433 | APL 23171 | M | 18.69 | 6.67 | 7.13 | 2.74 | 1.84 | 2.17 | 2.24 | 2.85 | 0.98 | 5.24 | 4.39 | 4.69 | 2.45 | 3.11 | 3.85 | 0.88 | 9.56 | 9.70 | 4.78 | 7.48 | 2.18 | 3.16 | 4.72 | 6.07 | 0.83 |
| INPAH 44434 | APL 23172 | M | 18.89 | 6.74 | 7.13 | 2.72 | 1.89 | 2.15 | 2.30 | 2.99 | 0.98 | 5.11 | 4.18 | 5.17 | 2.61 | 3.57 | 4.16 | 0.75 | 9.41 | 9.96 | 5.50 | 7.91 | 2.78 | 3.63 | 5.48 | 6.16 | 0.79 |
| INPAH 44435 | APL 23175 | M | 18.95 | 6.87 | 7.05 | 2.78 | 1.87 | 2.22 | 2.15 | 2.88 | 1.01 | 5.09 | 4.67 | 5.33 | 2.85 | 3.61 | 4.32 | 0.77 | 9.55 | 9.91 | 5.00 | 8.23 | 2.62 | 3.68 | 5.65 | 7.11 | 0.81 |
| INPAH 44436 | APL 23176 | F | 23.19 | 8.00 | 8.50 | 3.22 | 1.98 | 2.67 | 2.73 | 3.15 | 1.25 | 6.34 | 5.44 | 6.02 | 3.19 | 4.01 | 4.99 | 1.05 | 11.16 | 12.09 | 6.26 | 9.52 | 2.99 | 4.19 | 6.59 | 7.62 | 1.11 |
| INPAH 44437 | APL 23177 | F | 23.45 | 8.38 | 9.05 | 3.48 | 2.23 | 2.68 | 2.87 | 3.04 | 1.19 | 6.50 | 5.53 | 6.25 | 3.44 | 4.00 | 5.02 | 1.09 | 11.26 | 12.16 | 6.34 | 9.58 | 3.27 | 4.40 | 6.43 | 7.75 | 1.02 |
| MPEG 44636 | APL 23179 | F | 25.06 | 9.28 | 9.55 | 3.92 | 2.36 | 3.23 | 3.04 | 3.21 | 1.29 | 6.99 | 6.30 | 6.35 | 3.46 | 4.36 | 5.32 | 1.14 | 12.36 | 12.94 | 6.69 | 10.58 | 3.56 | 4.83 | 7.30 | 8.74 | 1.07 |
| MPEG 44637 | APL 23181 | M | 19.15 | 6.75 | 7.11 | 2.72 | 1.69 | 2.23 | 2.28 | 2.79 | 0.99 | 4.61 | 4.13 | 5.11 | 2.52 | 3.29 | 4.12 | 0.73 | 9.23 | 9.84 | 5.01 | 7.84 | 2.42 | 3.42 | 5.29 | 6.32 | 0.76 |
| MPEG 44638 | APL 23182 | F | 24.66 | 8.71 | 9.07 | 3.54 | 2.11 | 2.83 | 2.92 | 3.08 | 1.22 | 6.74 | 5.67 | 6.22 | 3.28 | 4.08 | 5.04 | 1.10 | 11.84 | 12.33 | 6.43 | 9.84 | 3.15 | 4.28 | 6.69 | 7.98 | 1.06 |
| MPEG 44639 | APL 23183 | M | 18.25 | 6.78 | 6.97 | 2.86 | 1.78 | 2.40 | 2.21 | 2.75 | 0.89 | 4.71 | 4.44 | 4.81 | 2.40 | 3.26 | 3.91 | 0.83 | 9.32 | 9.96 | 5.08 | 7.82 | 2.27 | 3.18 | 5.07 | 6.43 | 0.91 |
| MPEG 44640 | APL 23184 | F | 26.52 | 9.80 | 10.04 | 3.52 | 2.61 | 2.68 | 3.08 | 3.25 | 1.36 | 6.98 | 6.11 | 7.27 | 3.95 | 4.89 | 6.15 | 1.24 | 13.22 | 13.69 | 7.07 | 11.14 | 3.06 | 4.86 | 7.56 | 9.16 | 1.32 |
| MPEG 44641 | APL 23185 | M | 19.15 | 6.84 | 7.18 | 2.66 | 1.78 | 2.23 | 2.27 | 2.80 | 0.90 | 4.92 | 4.63 | 4.99 | 2.61 | 3.29 | 4.07 | 0.89 | 9.45 | 10.44 | 5.21 | 7.88 | 2.38 | 3.28 | 5.13 | 6.46 | 0.82 |
| MPEG 44634 | APL 22250 | M | 19.08 | 6.85 | 7.02 | 2.88 | 1.93 | 2.19 | 2.17 | 2.72 | 1.00 | 5.19 | 4.26 | 5.24 | 2.61 | 3.43 | 4.20 | 0.87 | 9.41 | 9.73 | 4.93 | 7.89 | 2.21 | 3.43 | 5.50 | 6.45 | 0.94 |
| MPEG 44635 | APL 22251 | M | 20.12 | 6.90 | 7.33 | 3.07 | 1.80 | 2.37 | 2.29 | 2.83 | 1.02 | 5.30 | 4.32 | 5.30 | 2.65 | 3.50 | 4.29 | 0.91 | 9.34 | 9.34 | 4.65 | 7.95 | 2.22 | 3.49 | 5.52 | 6.49 | 0.98 |
| INPAH 44439 | APL 22252 | M | 17.85 | 6.08 | 6.63 | 2.66 | 1.72 | 2.04 | 2.10 | 2.69 | 0.80 | 4.56 | 3.91 | 4.68 | 2.21 | 3.10 | 3.87 | 0.72 | 9.21 | 9.50 | 4.75 | 7.40 | 2.19 | 3.13 | 4.79 | 6.14 | 0.76 |
