# Supplementary material for: A new species of Pristimantis (Anura: Strabomantidae) from white-sand forests of central Amazonia, Brazil
Source: PeerJ. 2023 Jun 6;11:e15399. doi: 10.7717/peerj.15399 (PMC10252896; doi:10.7717/peerj.15399)
Supplement: Table S2 — Abbreviations: vouchers: INPAH, Instituto Nacional de Pesquisas da Amazônia; MPEG, Museu Paraense Emílio Goeldi; FNJV, Fonoteca Neotropical Jacques Vielliard; AT, air temperature (ºC); NN, number of notes per call; CD, call duration (ms); ND, note duration (ms); INI, inter-note interval (ms); LF, minimum frequency (Hz); HF, maximum frequency (Hz); and DF, dominant frequency (Hz). [file peerj-11-15399-s002.docx]

| Voucher | | AT | NN | CD | ND | INI | LF | HF | DF |
| --- | --- | --- | --- | --- | --- | --- | --- | --- | --- |
| specimen | call recorded |  |  |  |  |  |  |  |  |
| INPAH 44426 | FNJV 59105 | 25.8 | 7 | 601 | 23 | 74 | 2,686 | 3,756 | 3,295 |
| INPAH 44426 | FNJV 59105 | 25.8 | 6 | 626 | 24 | 72 | 2,787 | 3,785 | 3,316 |
| INPAH 44426 | FNJV 59105 | 25.8 | 7 | 606 | 25 | 72 | 2,724 | 3,825 | 3,338 |
| INPAH 44426 | FNJV 59105 | 25.8 | 6 | 550 | 21 | 87 | 2,682 | 3,796 | 3,316 |
| INPAH 44427 | FNJV 59106 | 25.9 | 8 | 710 | 27 | 70 | 2,932 | 4,287 | 3,725 |
| INPAH 44427 | FNJV 59106 | 25.9 | 7 | 682 | 29 | 80 | 2,892 | 4,281 | 3,768 |
| INPAH 44428 | FNJV 59107 | 25.6 | 6 | 665 | 25 | 86 | 2,957 | 5,241 | 3,919 |
| INPAH 44429 | FNJV 59108 | 25.6 | 6 | 626 | 25 | 82 | 3,064 | 4,391 | 3,790 |
| INPAH 44429 | FNJV 59108 | 25.6 | 7 | 704 | 28 | 84 | 2,947 | 4,361 | 3,704 |
| INPAH 44429 | FNJV 59108 | 25.6 | 8 | 779 | 22 | 86 | 2,977 | 4,391 | 3,682 |
| INPAH 44431 | FNJV 59109 | 25.5 | 6 | 593 | 16 | 99 | 3,018 | 4,495 | 3,876 |
| INPAH 44431 | FNJV 59109 | 25.5 | 6 | 564 | 13 | 97 | 2,911 | 4,513 | 3,919 |
| INPAH 44433 | FNJV 59110 | 24.8 | 8 | 747 | 22 | 81 | 2,804 | 4,419 | 3,639 |
| INPAH 44433 | FNJV 59110 | 24.8 | 8 | 761 | 23 | 83 | 2,785 | 4,456 | 3,575 |
| INPAH 44433 | FNJV 59110 | 24.8 | 8 | 746 | 26 | 77 | 2,660 | 4,432 | 3,531 |
| INPAH 44435 | FNJV 59111 | 25.3 | 7 | 644 | 29 | 74 | 2,729 | 4,415 | 3,575 |
| MPEG 44637 | FNJV 59112 | 24.4 | 7 | 748 | 40 | 69 | 2,789 | 4,386 | 3,424 |
| MPEG 44637 | FNJV 59112 | 24.4 | 5 | 613 | 38 | 87 | 2,706 | 4,548 | 3,359 |
| MPEG 44637 | FNJV 59112 | 24.4 | 6 | 650 | 37 | 75 | 2,773 | 4,700 | 3,402 |
| MPEG 44637 | FNJV 59112 | 24.4 | 6 | 691 | 39 | 78 | 2,763 | 4,721 | 3,402 |
| MPEG 44637 | FNJV 59113 | 24.4 | 7 | 738 | 31 | 84 | 2,758 | 4,768 | 3,424 |
| MPEG 44637 | FNJV 59113 | 24.4 | 7 | 712 | 35 | 75 | 2,695 | 4,773 | 3,359 |
| MPEG 44639 | FNJV 59114 | 24.2 | 6 | 649 | 17 | 109 | 3,035 | 5,113 | 3,790 |
| MPEG 44639 | FNJV 59114 | 24.2 | 10 | 1,061 | 20 | 95 | 3,020 | 5,280 | 3,661 |
| MPEG 44639 | FNJV 59115 | 24.2 | 9 | 974 | 24 | 95 | 2,905 | 5,053 | 3,725 |
| MPEG 44639 | FNJV 59115 | 24.2 | 5 | 596 | 31 | 98 | 3,176 | 4,544 | 3,747 |
